# A Spectrochemically Driven Study: Identifying Phenolic-Rich Extracts from *Helichrysum stoechas*, *Lavandula pedunculata*, and *Thymus mastichina* with Potential to Revert Skin Aging Effects

**DOI:** 10.3390/ph18121889

**Published:** 2025-12-14

**Authors:** Mário Pedro Marques, Euclides Landim, Carla Varela, Ricardo M. F. da Costa, Joana Marques, Luís A. E. Batista de Carvalho, Ana Silva, Maria Teresa Cruz, Rebeca André, Patrícia Rijo, Maria Inês Dias, Aida Carvalho, Paulo J. Oliveira, Célia Cabral

**Affiliations:** 1 Coimbra Institute for Clinical and Biomedical Research (iCBR), Clinic Academic Center of Coimbra (CACC), Faculty of Medicine, University of Coimbra, 3000-548 Coimbra, Portugal; silvamarques@student.uc.pt (M.P.M.); euclides.landim@student.uc.pt (E.L.); carla.varela@uc.pt (C.V.); 2 Center for Innovative Biomedicine and Biotechnology (CIBB), University of Coimbra, 3000-548 Coimbra, Portugal; anacrs@cnc.uc.pt (A.S.); trosete@ff.uc.pt (M.T.C.); pauloliv@cnc.uc.pt (P.J.O.); 3 Chemical Engineering and Renewable Resources for Sustainability (CERES), Faculty of Sciences and Technology, University of Coimbra, 3030-790 Coimbra, Portugal; 4 Molecular Physical-Chemistry R&D Unit, Department of Chemistry, University of Coimbra, LAQV/REQUIMTE, 3004-535 Coimbra, Portugal; rmfdcosta@uc.pt (R.M.F.d.C.); marques.jt@uc.pt (J.M.); labc@ci.uc.pt (L.A.E.B.d.C.); 5 Centre for Functional Ecology—Science for People and the Planet (CFE), Department of Life Sciences, University of Coimbra, 3000-456 Coimbra, Portugal; 6 CNC-UC, Center for Neuroscience and Cell Biology, University of Coimbra, 3004-504 Coimbra, Portugal; 7 Faculty of Pharmacy, University of Coimbra, 3000-548 Coimbra, Portugal; 8 Research Center for Biosciences and Health Technologies (CBIOS)—Universidade Lusófona, 1749-024 Lisbon, Portugal; rebeca.andre@ulusofona.pt (R.A.); patricia.rijo@ulusofona.pt (P.R.); 9 Centro de Química Estrutural, Institute of Molecular Sciences, Universidade de Lisboa, Campo Grande, 1749-016 Lisboa, Portugal; 10 Instituto de Investigação do Medicamento (iMed. ULisboa), Faculty of Pharmacy, University of Lisbon, 1649-003 Lisboa, Portugal; 11 Centro de Investigação de Montanha (CIMO), Laboratório Associado para a Sustentabilidade e Tecnologia em Regiões de Montanha (LA SusTEC), Instituto Politécnico de Bragança (IPB), Campus de Santa Apolónia, 5300-253 Bragança, Portugal; maria.ines@ipb.pt; 12 IPB, Campus de Santa Apolónia, 5300-253 Bragança, Portugal; acarvalho@ipb.pt; 13 CiTUR, Pólo Guarda, Av. Dr. Francisco Sá Carneiro 50, 6300-559 Guarda, Portugal; 14 Fundação Côa Parque, Rua do Museu, 5150-620 Vila Nova de Foz Côa, Portugal; 15 Faculty of Medicine, Instituto de Histologia e Embriologia, University of Coimbra, Rua Larga, Edifício da FMUC, Pólo 1, 2º Piso, 3004-504 Coimbra, Portugal

**Keywords:** plant extracts, phenolic compounds, skin aging, FTIR-ATR, HPLC-DAD-ESI/MS^n^

## Abstract

**Background/Objectives**: Plants inhabiting mediterranean-influenced climatic zones, like *Helichrysum stoechas* (L.) Moench subsp. *stoechas*, *Lavandula pedunculata* (Mill.) Cav., and *Thymus mastichina* (L.) L. subsp. *mastichina*, have been scarcely investigated regarding their richness in phenolic compounds, herein explored as sources of skin anti-aging compounds. **Methods**: In this investigation, Fourier transform infrared spectroscopy (FTIR) in attenuated total reflectance (ATR) mode and high-performance liquid chromatography coupled with diode-array detection and electrospray ionization tandem mass spectrometry (HPLC-DAD-ESI/MS^n^) were employed to chemically characterize the hydroethanolic extracts (HEs), and their cell-free antioxidant potential was screened. Thereafter, non-toxic concentrations of HEs were determined in human skin cells using Alamar blue^®^ and Sulforhodamine B assays. The cytoprotective and antioxidant effects of HEs were assessed in *tert*-butyl hydroperoxide-stimulated fibroblasts, their anti-inflammatory potential was studied in lipopolysaccharide-injured macrophages, and enzymatic inhibition assays were performed. Notably, the irritant effects of HEs were tested according to Test Guideline No. 439 of the Organization for Economic Co-operation and Development (OECD). **Results**: The major compounds identified in the *T. mastichina* and *L. pedunculata* HEs were rosmarinic and salvianolic acid derivatives, while *H. stoechas* HE was mainly composed of caffeoyl and feruloyl derivatives, and *O*-glycosylated flavonoids. *T. mastichina* (≤0.4 mg/mL) exhibited significant cytoprotective, anti-inflammatory, and antioxidant effects, as well as remarkable anti-hyaluronidase activity. **Conclusions**: Shedding light on the quantitative and qualitative chemical picture of these HEs highlighted *T. mastichina* as a promising candidate to target skin aging effects, which correlates with its phenolic content. Further investigation is warranted regarding its anti-aging pharmacological activity, which could lead to the development of plant-based skin anti-aging products.

## 1. Introduction

The skin contributes to 8 to 20% of the body’s weight and has an area of nearly 1.8 m^2^. The skin plays a crucial role in preventing electrolyte loss, regulating body temperature and evapotranspiration, and protecting against environmental hazards such as UV (ultraviolet) radiation and pollutants. The skin comprises three main layers: (1) the epidermis, mainly composed of keratinocytes at various differentiation stages; (2) the dermis, where fibroblasts are the major cells type; and (3) the hypodermis, mostly comprising adipocytes [1,2].

In addition to skin diseases, ranging from infectious and inflammatory conditions like acne and atopic dermatitis to cancerous conditions such as melanoma, there has been growing interest from the pharmaceutical and cosmetics industries in skincare. This has driven the development of skincare products aligned with sustainability guidelines [3,4]. From this perspective, today, consumers show a rising interest in products made with natural ingredients that may function in antioxidant, antiaging, and photoprotective capacities [2,3,5]. Although human skin cells possess defense mechanisms, such as protective enzymes like catalase and superoxide dismutase, an imbalance in the production of reactive oxygen species (ROS) and/or reactive nitrogen species (RNS), like nitric oxide (NO), may result in oxidative injury to proteins, lipids, and DNA. This oxidative damage can, in turn, result in cellular senescence or even cell death, processes that are often at the core of both skin aging and disease [6,7]. However, it is important to highlight that controlled production of RNS and ROS plays a crucial role in intracellular signaling [6], which is essential for maintaining normal cell physiology.

To this end, plants serve as a rich source of bioactive ingredients that can help counteract skin aging, particularly polyphenols such as flavonoids, tannins, and small phenolic acids. Their beneficial effects have been reported in human skin cells [8], namely as scavengers of ROS, or even as chelators of transition-metal ions [9]. Plants native to Mediterranean-influenced climatic zones are particularly intriguing, as they endure constant exposure to stressful edaphoclimatic conditions such as high temperatures, drought, and intense UV radiation. These challenges drive plants to synthesize a diverse arsenal of polyphenolic compounds that protect them from harmful photo-oxidative stress [9]. The Côa Valley, a region in northeastern Portugal, is home to numerous Paleolithic rock art sites that have been continuously discovered along the margins and hillsides of the Côa River in recent years. These sites are collectively preserved as the Côa Valley Archaeological Park and have held UNESCO World Heritage status since 1998. This territory is characterized by scarce rainfall and extreme summer temperatures, which can exceed 40 °C on extremely hot summer days [10]. This Mediterranean-influenced climatic zone gives rise to outstanding flora that has been studied by members of our research team [10,11,12,13], and from which *Helichrysum stoechas* (L.) Moench subsp. *stoechas* (Asteraceae), *Lavandula pedunculata* (Mill.) Cav. (Lamiaceae), and *Thymus mastichina* (L.) L. subsp. *mastichina* (Lamiaceae) were selected in this study. The criteria for their selection included their emblematic presence in the Côa Valley landscape, their ethnomedicinal use in treating skin conditions, and their cultural relevance to the local community. These factors highlight their significance and underscore the importance of their valorization [10]. Furthermore, throughout the rest of the Iberian Peninsula and even in other Mediterranean territories, these plants are reported to be traditionally used for treating skin diseases. *L. pedunculata* [14], and specifically *T. mastichina*, has been reported to be used in the treatment of acne, acting as an antiseptic and anti-inflammatory when applied on the skin [15], being beneficial for the treatment of skin injuries [16]. Also, noteworthy is the traditional application of both *H. stoechas* [17] and *T. mastichina* [15] as air-fresheners and for homemade cosmetics. However, plants like *L. pedunculata* and *T. mastichina*, the latter an Iberian endemism, still lack detailed scientific studies, namely regarding chemical elucidation of their phenolic-rich extracts, possibly due to their limited geographical distribution [15,18].

Recent research on plants from the genus *Helichrysum*, including *H. stoechas*, has revealed increasingly complex chemical profiles, helping not only to confirm the biological activities of its extracts but also to identify the compounds responsible for these effects. Major groups of bioactive constituents include flavonoids like kaempferol and quercetin derivatives; phenolic acids such as chlorogenic; caffeic and ferulic acid derivatives; and essential oils and terpenoids [19,20,21,22,23]. This rich chemical composition underlies a broad range of bioactivities reported across *Helichrysum* species, including antioxidant, anti-inflammatory, anticancer, and neuroprotective effects. Notably, compounds from the caffeoylquinic acid group have positioned these plants as promising sources of antiviral agents, and their significant antibacterial and antifungal properties are also well documented [19].

Beyond the widely studied essential oils from plants of the *Lavandula* genus, the diversity of non-volatile compounds also makes plants like *L. pedunculata* a valuable source of pharmacologically relevant phytochemicals [18]. Major phenolic acids include rosmarinic, caffeic, and salvianolic B acids, while luteolin derivatives and eriodictyol-*O*-glucuronide are among the principal flavonoids reported [5,18,24,25]. *Lavandula* extracts have demonstrated notable antioxidant activity, broad-spectrum antimicrobial effects, anti-inflammatory and anticancer properties, and other beneficial activities [18].

Similarly, the essentials oils from *Thymus* have been widely investigated, more so than phenolic-rich extracts [26,27]. Even though extracts obtained from *T. mastichina* have been found to contain a wide range of bioactive compounds, including 2- and 3-methoxysalicylic acids, apigenin, caffeic acid, chlorogenic acid, kaempferol, luteolin, quercetin, rosmarinic acid, sakuranetin, sterubin, salvianolic acid derivatives, and glycosidic derivatives [27,28,29,30], studies have also highlighted the biological activities of its essential oil and extracts, including antibacterial, antifungal, antioxidant, insecticidal, repellent, antiviral, anti-Alzheimer, and anti-inflammatory effects. Its antimicrobial properties make it a promising candidate for natural antimicrobial agents, while its antioxidant activity provides an attractive alternative to synthetic antioxidants [27]. Altogether, these attributes give plants of the *Helichrysum*, *Lavandula*, and *Thymus* genera the potential for diverse applications in pharmaceuticals, food additives, cosmetics, or even as phyto-pharmaceutics.

Investigating the chemical composition and the pharmacological potential of plant extracts is a crucial step in the sustainable valorization of natural products. In an agroforestry context, for example, adding value to non-cultivated plant species can create economic incentives for improved land management practices [31]. Similarly, in pharmaceutical, nutraceutical, and cosmetic applications, characterizing bioactive compounds and evaluating how their concentrations fluctuate throughout the seasons and years can help unlock their commercial potential for novel plant-based products [32]. These examples illustrate the broader relevance of such studies in promoting the use of natural sources within sustainable green chemistry and circular economic frameworks.

Therefore, in the present work, hydroethanolic extracts (HEs) were prepared from *H. stoechas*, *L. pedunculata*, and *T. mastichina*, and chemically characterized in detail. Subsequently, the cell-free antioxidant activity of the HEs was screened, and their cytotoxic effects were examined in a panel of normal human skin cells to ensure the safety of extracts upon potential skin application. Furthermore, their cytoprotective properties, effects on oxidative stress, potential anti-inflammatory activity, and cell-free inhibitory activity toward skin-aging-related enzymes were assessed. The irritant effect of the most promising HE was tested in compliance with OECD Test Guideline No. 439 [33]. Overall, this pioneering study highlights the potential of these plants as a promising source of bioactive ingredients, as demonstrated simultaneously by the consortium established between FTIR-ATR and HPLC-DAD-ESI/MS^n^, paving the way for the development of innovative plant-based products capable of targeting human skin aging effects.

## 2. Results

### 2.1. FTIR-ATR Spectroscopy

**Figure 1:**
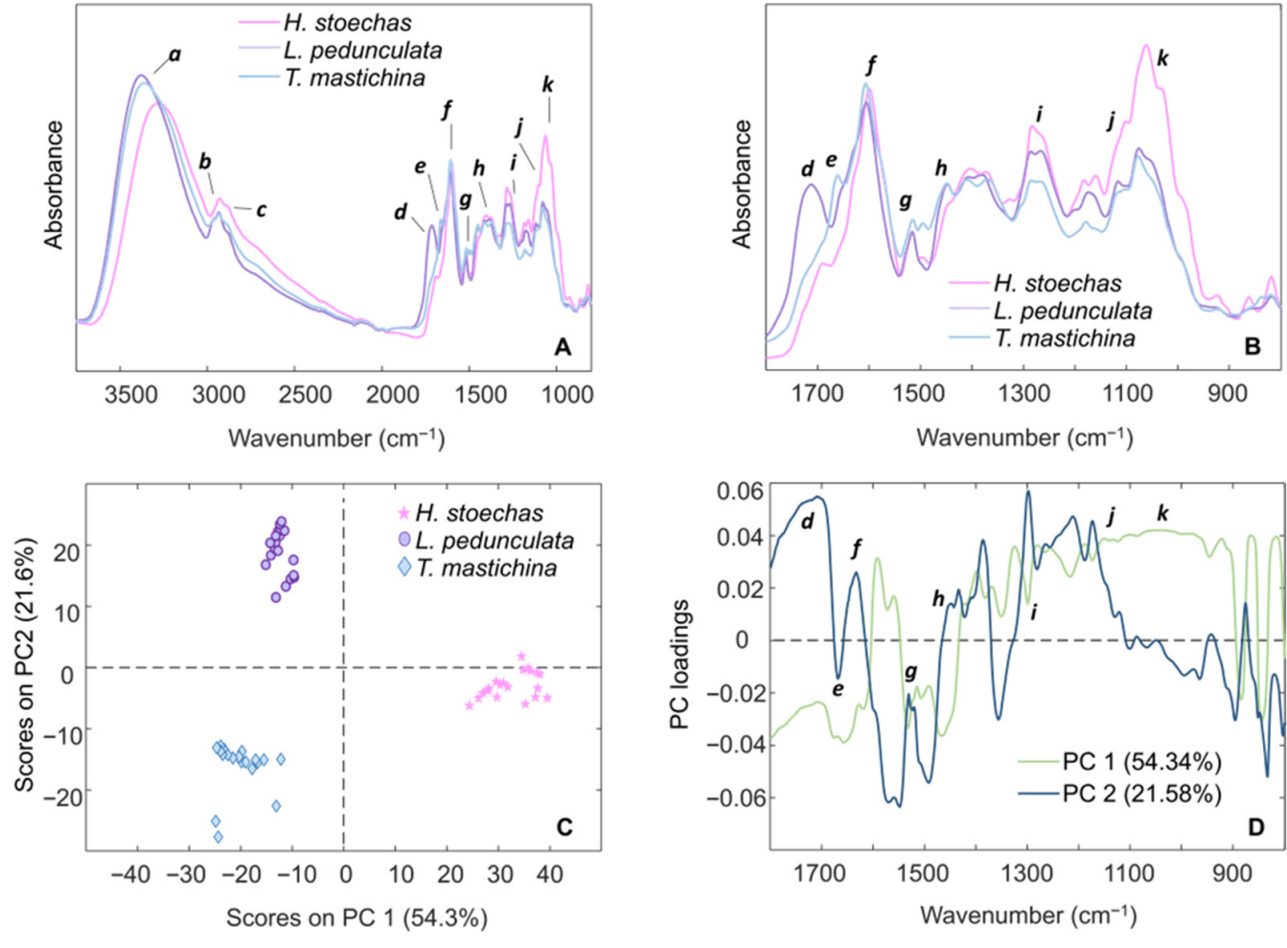
(**A**) Mean FTIR-ATR spectra in the range of 3700–800 cm^−1^; (**B**) mean FTIR-ATR spectra in the region of 1800–800 cm^−1^; (**C**) plot of principal component one (PC1) and principal component two (PC2) scores of *H. stoechas*, *L. pedunculata*, and *T. mastichina* HEs; and (**D**) loading plot for PC1 and PC2. Relevant spectral range assignments are represented by lowercase italicized letters (a–k) as summarized in Table 1.

As a driving force for this investigation, different spectra were collected in the range of 3700–800 cm^−1^ (Figure 1A), and IR bands were assigned (Table 1). This approach revealed that the HEs were particularly distinct in the region of 1800–800 cm^−1^ (Figure 1B).

Principal Component Analysis (PCA) was applied to the FTIR-ATR data to investigate the chemometric relationships between the samples, revealing distinct clusters along PC1 (explaining 54.3% of the variance) and PC2 (21.6%) (Figure 1C). In what concerns the primary axis of separation between the spectral clusters, the HE from *H. stoechas* capitula was positioned on the positive side of PC1. Conversely, a second cluster, comprising the HEs of *L. pedunculata* and *T. mastichina*, was located on the negative side of PC1. This clustering indicates a greater compositional similarity between *L. pedunculata* and *T. mastichina* compared to *H. stoechas*. Secondly, along PC2 (21.6%), the spectra of *L. pedunculata* were distinctly separated from those of the other two species, occupying the positive coordinates of PC2. This distinct sample segregation, supported by the interpretation of the corresponding principal component loadings plot (Figure 1D), provides valuable insights into the relative compositional differences between the HEs of the studied plant species.

**Table 1:** FTIR-ATR wavenumbers and assignments of functional groups for the HEs in this study.

Region (cm^−1^)	Assignment	References	Hs	Lp	Tm
*a* 3382–3278	*ν*(O–H)	[34,35]	3278	3382	3367
*b* 2935–2930	*ν*_antisym_(CH_3_ and CH_2_), aliphatic compounds	[36,37]	2930	2935	2935
*c* 2885–2881	*ν*_sym_(CH_3_ and CH_2_), aliphatic compounds	[38,39]	2884	2881	2885
*d* 1714–1690	*ν*(C=O) in COOH	[36,37,40]	1690	1713	1714
*e* 1661–1660	*ν*(C–C) aromatic ring, *ν*(C=O) in COOH	[34,35,40]	–	1661	1660
*f* 1606–1599	*ν*(C=C) aromatic ring	[34,36,40]	1599	1606	1606
*g* 1517–1515	*ν*(C–C) aromatic ring	[34,35,39]	1516	1517	1515
*h* 1497–1446	*δ*(CH_3_ and CH_2_) aliphatic compounds, polysaccharides; *ν*(C–C) aromatic ring	[35,36,37]	1497w; 1446	1494sh; 1449	1495vw; 1449
*i* 1286–1264	*δ*(C–H), *ν*(C–OH)	[34,39]	1283; 1264sh	1286sh; 1267	1285; 1267sh
*j* 1175–1101	*ν*(C–O–C) ester, *ν*(C–O) and *δ*(C–OH) carbohydrates	[36,37]	1160; 1101	1175; 1117	1179; 1102
*k* 1077–1033	*ν*(C–O) and *ν*(C–C) carbohydrates	[37,38]	1062; 1033sh	1077; 1051sh	1077; 1051sh

Lowercase italicized letters (a–k) represent the IR bands assigned in the spectra. Abbreviations: antisym, antisymmetric; sym, symmetric; ν, stretching; δ, bending; sh, shoulder; w, weak; vw; very weak; Hs, *H. stoechas*; Tm, *T. mastichina*; and Lp, *L. pedunculata*.

### 2.2. Total Phenolic and Total Flavonoid Contents and Cell-Free Antioxidant Activity

**Figure 2:**
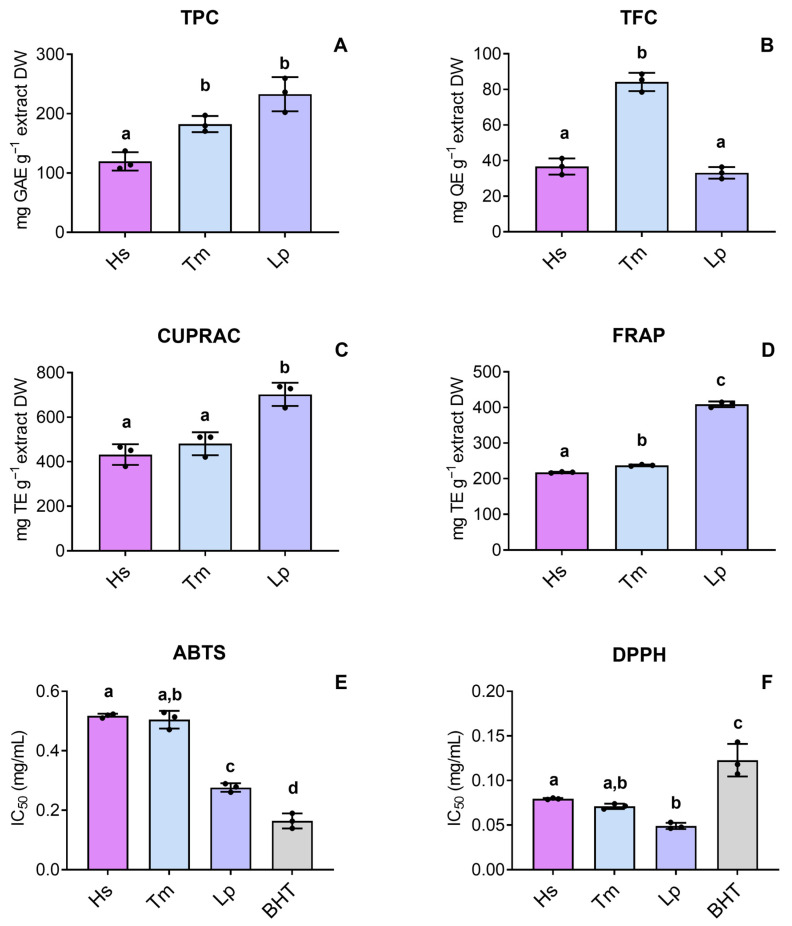
(**A**) Total phenolic content (TPC) (mg GAE g^−1^ extract DW); (**B**) total flavonoid content (TFC) (mg QE g^−1^ extract DW); (**C**) cupric (CUPRAC) and (**D**) ferric (FRAP) reducing powers (mg TE g^−1^ extract DW). Free-radical-scavenging activity of (**E**) ABTS and (**F**) DPPH (IC_50_ values, mg/mL of HE) using BHT (butylated hydroxytoluene) as a positive control. Bars represent the mean ± SD of three independent experiments performed in triplicate. Different superscript letters (a–d) indicate significant differences between groups. The statistical analysis was carried out by one-way ANOVA, followed by Tukey’s post hoc test (*p* < 0.05). Abbreviations: Hs, *H. stoechas*; Tm, *T. mastichina*, and Lp, *L. pedunculata*.

Regarding total phenolic content (TPC)*, L. pedunculata* and *T. mastichina* showed higher amounts in comparison to *H. stoechas* (Figure 2A), while *T. mastichina* stood out by showing the highest total flavonoid content (TFC) (84.176 ± 5.149 quercetin equivalents (QE) g^−1^ extract dry weight (DW)) (Figure 2B). Complementary information on the extraction yields (%) of the selected plant material is summarized in Table S1 (Supplementary Materials). Also, the tested HEs were more effective in terms of cupric ion reducing antioxidant capacity (CUPRAC) than ferric reducing antioxidant power (FRAP) (Figure 2C and Figure 2D, respectively), with substantially higher CUPRAC activity, ranging between 431.868 ± 46.317 trolox equivalents (TE) g^−1^ extract DW in *H. stoechas* to 702.593 ± 52.427 TE g^−1^ extract DW in *L. pedunculata*. Also, HEs presented a strong ability to inhibit the 2,2-Diphenyl-1-picrylhydrazyl radical (DPPH^•^) (Figure 2F), even outperforming the reference antioxidant butylated hydroxytoluene (BHT; IC_50_ = 0.123 ± 0.018 mg/mL). However, regarding the 2,2′-azino-bis(3-ethylbenzothiazoline-6-sulfonic acid) radical cation (ABTS^•+^) assay, HEs did not show the same potential (Figure 2E). The determined values (mean ± SD) in these assays are summarized in Table S2 (Supplementary Materials).

### 2.3. HPLC-DAD-ESI/MS^n^ Analysis

**Figure 3:**
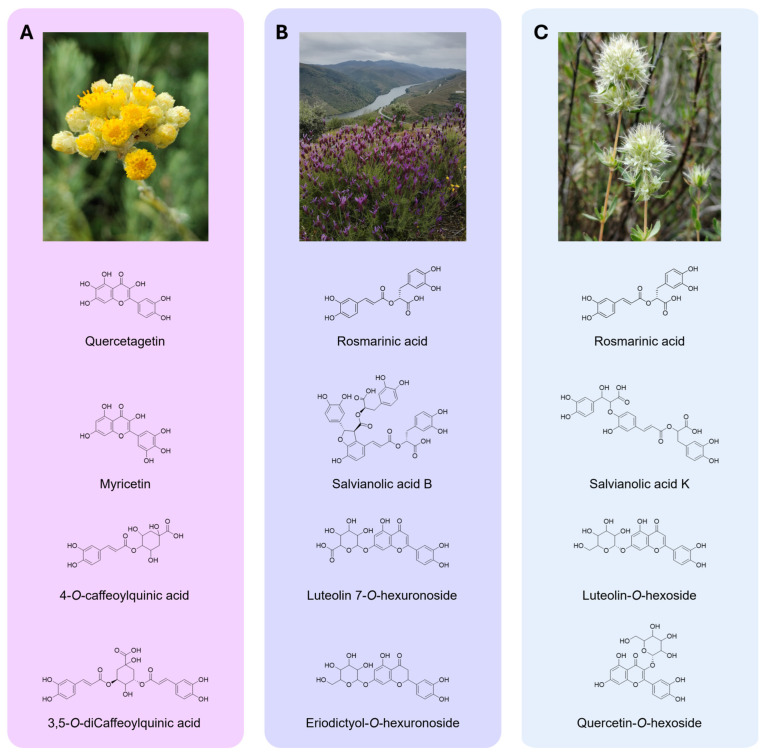
Plants harvested in Côa Valley (Portugal) and chemical structures of the major phenolic compounds identified in the studied extracts. (**A**) Capitula of *H. stoechas*; (**B**) flowering aerial parts of *L. pedunculata* and (**C**) *T. mastichina*. Photographs of plants were captured by Mário Pedro Marques. Chemical structures were designed using ChemDraw Software v.14.0.

Among the samples studied, 82 different compounds were tentatively identified, including 30 in the HE from *H. stoechas*, 29 in *L. pedunculata*, and 23 in the *T. mastichina* sample. The chromatographic and mass responses, as well as respective tentative identification of the phenolic compounds found, are described in Table 2. The chemical structures of the major phenolics identified in HE are illustrated in Figure 3. The illustrative phenolic profiles recorded at 280 and 370 nm are presented in Figure S2 (Supplementary Materials).

Regarding phenolic acid derivatives in the HE from *H. stoechas*, caffeoyl and feruloyl derivatives of quinic acid were found and tentatively identified as 1-*O*-, 4-*O*-, and 5-*O*-caffeoylquinic acids (peaks 1*^Hs^*/2*^Hs^*/3*^Hs^*); 3,5-*O*-, 3,4-*O*-, and 4,5-*O*-dicaffeoylquinic acids (peaks 15*^Hs^*/17*^Hs^*/18*^Hs^*); and 5-*O*-feruloylquinic acid (peak 5*^Hs^*). For instance, peak 20*^Hs^* ([M-H]^−^ at *m*/*z* 601) was tentatively identified as malonyl-dicaffeoyl-quinic acid, and peak 4*^Hs^* ([M-H]^−^ at *m*/*z* 179) was identified as caffeic acid. Undoubtedly, *O*-glycosylated flavonoids stood out in this HE, especially isorhamnetin derivatives (peaks 19*^Hs^*/22*^Hs^*/29*^Hs^*), kaempferol-*O*-glycosides assigned by peaks 27*^Hs^*, 28*^Hs^* and 30*^Hs^*, and the peak 23*^Hs^* was attributed to luteolin-*O*-dipentoside. The extract also contains myricetin derivatives, as demonstrated by the peaks 6*^Hs^* and 24*^Hs^*, in addition to quercetin *O*-glycosylated derivatives linked to phenolic acid moieties, associated with peaks 25*^Hs^* and 26*^Hs^* ([M-H]^−^ at *m*/*z* 609 and 695, respectively). Peaks 14*^Hs^* and 16*^Hs^* ([M-H]^−^ at *m*/*z* 549) were identified as quercetin-*O*-malonyl-hexoside isomers I and II, and the peak 13*^Hs^* ([M-H]^−^ at *m*/*z* 463) was identified as quercetin-3-*O*-glucoside. Lastly, quercetagetin derivatives at peaks 7*^Hs^*, 8*^Hs^*, and 10*^Hs^* accounted for the complex flavonoids composition of this extract, unlike peaks 9*^Hs^*, 11*^Hs^*, and 12*^Hs^* ([M-H]^−^ at *m*/*z* 461), for which it was not possible to clearly distinguish between quercetagetin and mirycetin derivatives.

For instance, *L. pedunculata* showed a miscellaneous phenolic acid composition according to the identification of caffeic acid derivatives 1*^Lp^* ([M-H]^−^ at *m*/*z* 341) and 2*^Lp^* ([M-H]^−^ at *m*/*z* 489), some *p*-Coumaroyl hexoside isomers (peaks 3*^Lp^*/4*^Lp^*/7*^Lp^*), and others, such as 11*^Lp^* ([M-H]^−^ at *m*/*z* 521), 14*^Lp^* ([M-H]^−^ at *m*/*z* 719), and 21*^Lp^* ([M-H]^−^ at *m*/*z* 717), tentatively identified as rosmarinic acid hexoside, sangerinic acid, and salvianolic acid B. Notwithstanding, *O*-glycosylated derivatives also accounted for a substantial part of the composition, such as those derived from apigenin algycones (peaks 16*^Lp^*/17*^Lp^/*22*^Lp^*), luteolin (peaks 12*^Lp^*/13*^Lp^/*18*^Lp^/*23*^Lp^/*26*^Lp^*), methylluteolin (peak 19*^Lp^* and peak 20*^Lp^*), and eriodyctiol (peak 10*^Lp^*). Interestingly, peak 5*^Lp^*, identified as medioresinol and presenting a deprotonated ion [M-H]^−^ at *m*/*z* 387, belongs to a different class of compounds, the lignans.

Similarly to *L. pedunculata*, a variety of phenolic acids were found in the HE from *T. mastichina*, including 4-*O*-caffeoylquinic acid (peak 1*^Tm^*); hydroxyjasmonic acid (peak 2*^Tm^*); caffeic acid (peak 5*^Tm^*); rosmarinic acid (peak 19*^Tm^*), including a glycosylated form (peak 12*^Tm^*); salvianolic acid K isomers represented by the peaks 17*^Tm^*, 18*^Tm^*, and 20*^Tm^*, showing a deprotonated ion [M-H]^−^ at *m*/*z* 555; and salvianolic acid B/E (peak 22*^Tm^*, [M-H]^−^ at *m*/*z* 717). According to this analysis, flavonoid composition is varied and complex, with plenty of luteolin-*O*-glycosylated forms being found, namely three isomers (peaks 10*^Tm^*, 14*^Tm^* and 15*^Tm^*, [M-H]^−^ at *m*/*z* 447) and peaks 28*^Tm^* and 29*^Tm^*. Similarly, eriodyctiol-*O*-hexoside isomers I, II, and III (peaks 6*^Tm^*, 7*^Tm^*, and 16*^Tm^*, [M-H]^−^ at *m*/*z* 449) were tentatively identified, in addition to several quercetin and *O*- and *C*-glycosylated apigenin forms, as well as naringenin and chrysoeriol derivatives.

### 2.4. Cytotoxic Effects on Normal Skin Cells

**Figure 4:**
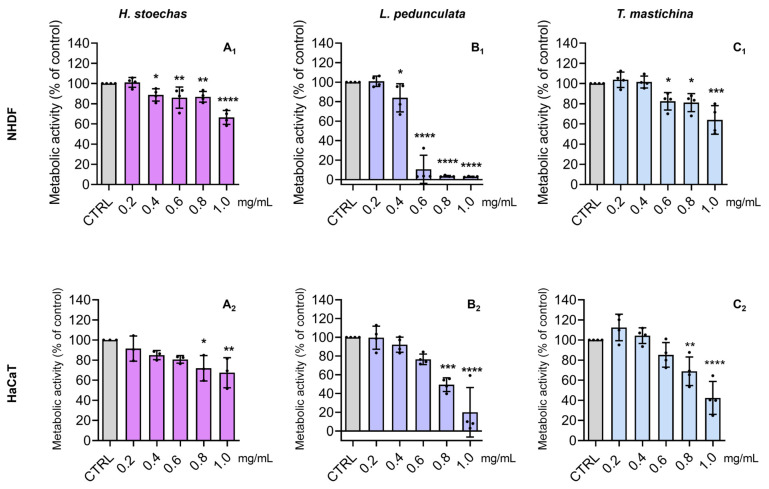
Effect of the HE of *H. stoechas* (**A_1_**,**A_2_**), *L. pedunculata* (**B_1_**,**B_2_**), and *T. mastichina* (**C_1_**,**C_2_**) on the metabolic activity of NHDF and HaCaT cells, respectively. Cells were treated with HE (0.2–1.0 mg/mL) for 24 h, and metabolic activity was evaluated by the Alamar blue^®^ assay. Untreated cells were used as control (CTRL). The results are expressed as a percentage (%) of metabolic activity relative to the CTRL and represent the mean ± SD of four independent experiments, each one performed in triplicate. The statistical analysis was carried out by one-way ANOVA followed by Dunnett’s multiple comparisons test (* *p* < 0.05, ** *p* < 0.01, *** *p* < 0.001, and **** *p* < 0.0001 versus CTRL).

**Figure 5:**
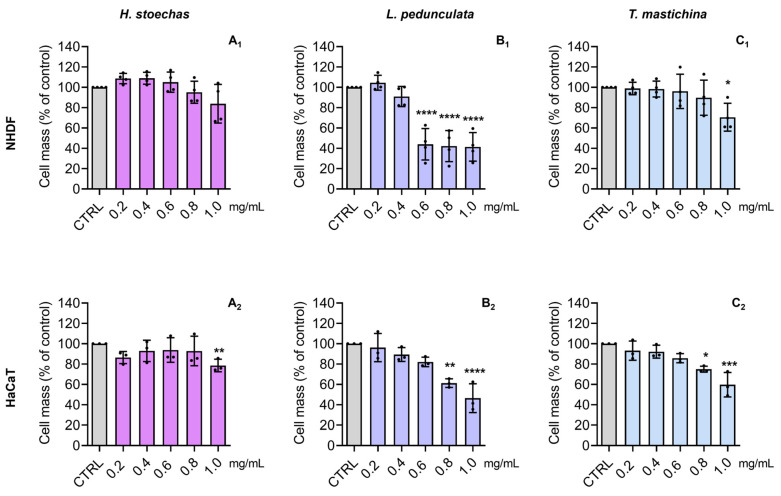
Effect of the HE of *H. stoechas* (**A_1_**,**A_2_**), *L. pedunculata* (**B_1_**,**B_2_**), and *T. mastichina* (**C_1_**,**C_2_**) on the cell mass of NHDF and HaCaT cells, respectively. Cells were treated with HE (0.2–1.0 mg/mL) for 24 h, and cell mass was evaluated by the SRB assay. Untreated cells were used as the control (CTRL). The results are expressed as a percentage (%) of cell mass relative to the CTRL and represent the mean ± SD of four independent experiments, each one performed in triplicate. The statistical analysis was carried out by one-way ANOVA followed by Dunnett’s multiple comparisons test (* *p* < 0.05, ** *p* < 0.01, *** *p* < 0.001, and **** *p* < 0.0001 versus CTRL).

NHDF cells have been successfully used by our research team [41] as a suitable toxicological model for evaluating new molecules for cosmetics [7]. Moreover, several authors have also used skin fibroblasts to evaluate the effects of plant-derived phenolics [8]. The HaCaT cells, which share similar differentiation features to the ones exhibited by in vivo keratinocytes [42], are also a suitable model for cytotoxic effects assessment. Therefore, the cytotoxic effect of HEs (0.2–1.0 mg/mL) on metabolic activity (Figure 4) and cell mass (Figure 5) was tested in two normal skin cell lines (NHDF and HaCaT). In general, all HEs presented a dose-dependent effect on cell metabolic activity, with no significant decrease at concentrations ≤ 0.2 mg/mL. For instance, in terms of metabolic activity performance, the HE from *H. stoechas* at 1 mg/mL caused a decrease of about 20–40% in comparison to the control, in both NHDF and HaCaT cells (Figure 4A_1_ and Figure 4A_2_, respectively), and a 20% decrease in cell mass (Figure 5A_1_ and Figure 5A_2_, respectively). For instance, the extract of *L. pedunculata* evoked the largest cytotoxic effect, namely regarding NHDF metabolic activity (Figure 4B_1_), reducing it to about 80 to 100% at ≥0.6 mg/mL. This dose-dependent effect of *L. pedunculata* was also observed for cell mass determination (Figure 5B_1_). Nevertheless, the cytotoxic effect on HaCaT cells was less pronounced (Figure 4B_2_ and Figure 5B_2_). Concerning the HE of *T. mastichina*, at ≤0.4 mg/mL this extract did not significantly affect the cell mass or impair the cell metabolic activity of either NHDF or HaCaT cell lines (Figure 4C_1_ and Figure 4C_2_, and Figure 5C_1_ and Figure 5C_2_, respectively).

### 2.5. Antioxidant and Cytoprotective Effects

**Figure 6:**
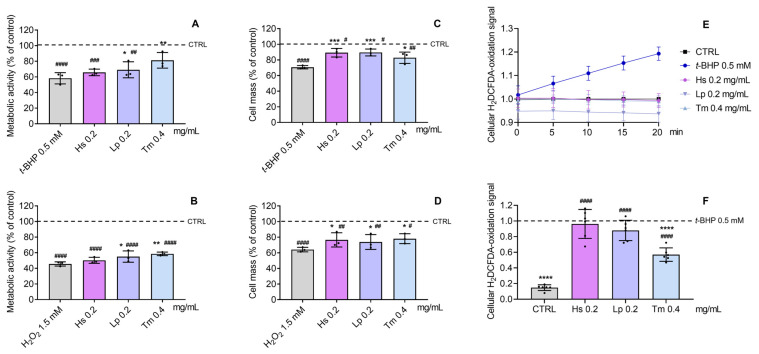
Cytoprotective effect of HE against *t*-BHP (**A**,**C**) and H_2_O_2_-induced (**B**,**D**) metabolic activity and cell mass decrease, respectively, in NHDF cells pre-incubated for 24 h with HE. Cells were subsequently incubated with *t*-BHP (0.5 mM) or H_2_O_2_ (1.5 mM) for 3 h. (**E**) Monitoring of the pro-oxidant effect of *t*-BHP in comparison to the HE-treated and CTRL NHDF cells. (**F**) Protective effect against *t*-BHP-induced oxidative stress in NHDF, pre-incubated for 24 h with HEs. Untreated cells were used as the CTRL (control). The effect on cell metabolic activity and cell mass was evaluated by the Alamar blue^®^ and SRB assays, respectively. Cellular oxidative stress was determined by the H_2_DCFDA assay. Results represent mean ± SD of three (**A**–**D**) and six (**E**,**F**) independent experiments, performed in triplicate. Statistical analysis was performed by one-way ANOVA followed by Dunnett’s multiple comparisons test (* *p* < 0.05, ** *p* < 0.01, *** *p* < 0.001, **** *p* < 0.0001 versus *t*-BHP and/or H_2_O_2_; ^#^ *p* < 0.05, ^##^ *p* < 0.01, ^###^ *p* < 0.001, ^####^ *p* < 0.0001 versus CTRL). Abbreviations: *t*-BHP, *tert*-butyl hydroperoxide; H_2_O_2_, hydrogen peroxide; H_2_DCFDA, 2′,7′-dichlorodihydrofluorescein diacetate; Hs, *H. stoechas*; Tm, *T. mastichina*; and Lp, *L. pedunculata*.

According to our results (Figure 6), *t*-BHP (0.5 mM) and H_2_O_2_ (1.5 mM) effectively promoted nearly a 40% and a 60% cell metabolic activity decrease, respectively, in comparison to CTRL, and the HE of *L. pedunculata* (* *p* < 0.05) and *T mastichina* (** *p* < 0.01) successfully counteracted this effect, whereas the extract from *H. stoechas* was ineffective (Figure 6A,B). Concerning the reduction in cell mass induced by *t*-BHP and H_2_O_2_ (Figure 6C,D), all extracts were able to prevent this loss. This cytoprotective effect warranted a more detailed investigation of how the extracts influence cellular oxidative stress, which was subsequently assessed using the H_2_DCFDA assay. As such, the absence of pro-oxidant effects in these samples were confirmed in the HEs-treated cells, in comparison to the increase in H_2_DCFDA-oxidation signals promoted by *t*-BHP (Figure 6E). Furthermore, regarding the HEs’ modulation of oxidative stress (Figure 6F), the promising effect of *T. mastichina* (**** *p* < 0.0001) in the protection of NHDF cells against the oxidative stress promoted by *t*-BHP was confirmed, while *L. pedunculata* and *H stoechas* did not prevent oxidative stress in *t*-BHP-treated cells.

### 2.6. Effect on the Levels of Nitrites

**Figure 7:**
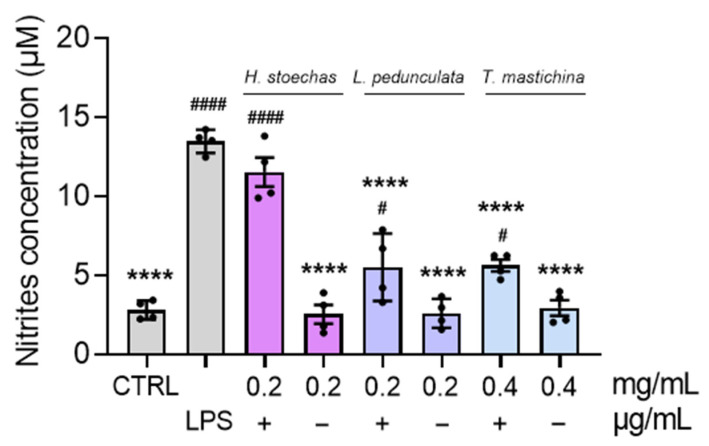
Effect of HEs on LPS-stimulated RAW 264.7 macrophages. The cells were treated with the highest non-toxic concentration of HE, in the absence (−) and presence (+) of 0.1 µg/mL LPS, for 24 h. Untreated cells were used as the control (CTRL). The results are expressed as nitrite concentration (µM) and represent the mean ± SD of four independent experiments, each one performed in triplicate. Statistical analysis was performed by one-way ANOVA followed by Dunnett’s and Šidák’s multiple comparisons tests (**** *p* < 0.0001 versus LPS) and (^#^ *p* < 0.05 and ^####^ *p* < 0.0001 versus CTRL).

The anti-inflammatory potential of HEs, at the highest non-toxic concentrations, was investigated by determining levels of nitrites in LPS-activated macrophages (Figure 7). Therefore, macrophages were subjected to HE treatment without (−) LPS stimulation, showing the absence of pro-inflammatory effects on these cells, with production of nitrites comparable to that in the CTRL cells, as well as with LPS stimulation (+) to unveil its potential anti-inflammatory effects. A cytotoxic assessment of these HEs on RAW 264.7 cells was also carried out (Supplementary Materials, Figure S3). Overall, the results demonstrated a highly significant reduction (**** *p* < 0.0001) in nitrite production when macrophages were treated with *L. pedunculata* and *T. mastichina*. Both extracts reduced the levels of nitrites to less than half of those observed in LPS-stimulated cells. In contrast, the extract of *H. stoechas* did not reduce nitrite levels (compared to LPS), suggesting that it does not exert an anti-inflammatory effect.

### 2.7. Cell-Free Evaluation of Enzyme Inhibitory Activity

The efficacy of HEs (at the highest non-toxic concentration for normal skin cells) in inhibiting the activity of four enzymes associated with skin aging was investigated using cell-free systems (Table 3). The most notable result was obtained with the *T. mastichina* extract, which demonstrated a strong hyaluronidase inhibition of approximately 90%, comparable to the inhibition achieved by the positive control, EGCG. *L. pedunculata* also showed remarkable potential, reaching almost 80% of hyaluronidase’s inhibition. Regarding tyrosinase activity, all extracts showed a considerable capacity to inhibit this enzyme (around 60%), despite none of them being able to reach the effect of the positive control kojic acid. Similarly, the activity of the tested samples did not outperform the capacity of EGCG to inhibit elastase, with *L. pedunculata* HE showing no activity. Regarding acetylcholinesterase’s inhibition, only *T. mastichina* presented reduced potential (around 30%), while no effect was detected for *L. pedunculata* and *H. stoechas*.

### 2.8. Skin Irritation Effects

**Figure 8:**
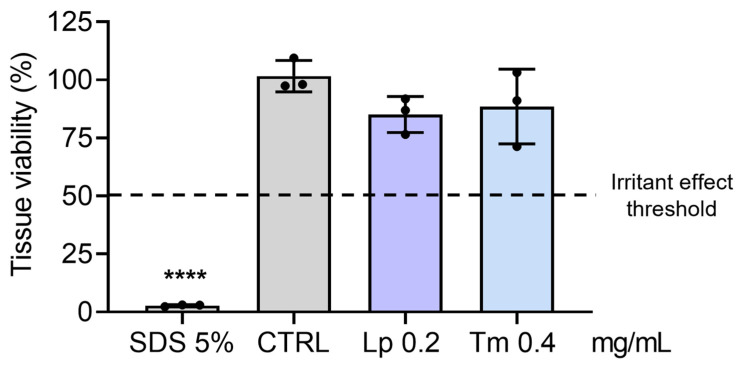
Skin irritation test. HEs irritancy was evaluated in a 3D reconstructed human epidermis model (SkinEthic™ RHE). The inserts were treated for 42 min in the absence (control—CTRL) and in the presence of HEs or with 5% (*w*/*v*) sodium dodecyl sulfate (SDS, an irritant used as a positive control). Tissue viability was assessed by the MTT assay. The results are the mean ± SD of three independent tissues, and tissue viability is expressed as a % of the CTRL (tissue exposed to PBS). The statistical analysis was performed by one-way ANOVA, followed by Dunnett’s and multiple comparisons tests (**** *p* < 0.0001 versus CTRL). Abbreviations: Tm, *T. mastichina*; Lp, *L. pedunculata*.

Considering the promising results obtained in this study with extracts from *L. pedunculata* and *T. mastichina*, their potential to evoke skin irritation was evaluated in a reconstructed human epidermis 3D model (SkinEthic™ Reconstructed Human Epidermis), in compliance with OECD Test Guideline No. 439. A substance is considered non-irritant if it does not reduce tissue viability to ≤50% (as observed for SDS, used as positive control) [33]. The results demonstrated that the tested HE did not display an irritating effect, since the tissues exposed to both extracts presented tissue viability higher than 50% (Figure 8).

## 3. Discussion

In this study, we aimed to assess the phenolic-rich extracts of plants inhabiting Mediterranean-influenced climatic zones, such as *H. stoechas*, *L. pedunculata*, and *T. mastichina*, as sources of ingredients to counteract human skin aging. To achieve this, we established a spectrochemically driven study by first characterizing their chemical profiles through FTIR-ATR and then by HPLC-DAD-ESI/MS^n^, and investigated their role in modulating oxidative and inflammatory processes, which are key factors in preventing skin aging. The key focus of this analysis was on hydroethanolic extracts (80:20%, *v*/*v*), due to their effectiveness in extracting a broad spectrum of bioactive constituents, including both polar and non-polar compounds [12].

The utilization of plant-based extracts for cosmetic, pharmaceutical, or nutraceutical applications relies on a thorough understanding of their qualitative and quantitative variations. As such, FTIR-ATR has emerged as a technique for the rapid characterization of large numbers of plant-derived samples, owing to its high-throughput capacity, sensitivity, non-targeted, and non-destructive nature [32,43]. Accordingly, FTIR-ATR analysis revealed a prominent band around 1714–1690 cm^−1^ (*d*) in *L. pedunculata* HE, which was scarcely observed in the spectra of *H. stoechas* and *T. mastichina*. This band was ascribed to the frequency of the C=O bond stretching vibration in carboxylic groups (COOH) [36,37,40]. The presence of this feature, along with the prominence of bands associated with structural features of aromatic rings, namely bands *e*, *f*, and *g*, suggest a relatively higher phenolic acid content in the HE of *L. pedunculata* compared to *T. mastichina* and *H. stoechas*. Indeed, this conclusion was further supported by the HPLC-DAD-ESI/MS^n^ data, which confirmed a higher abundance of phenolic acid derivatives in *L. pedunculata*.

Nevertheless, the overall extraction yield of the HE obtained from the flowering aerial parts of *L. pedunculata* (10.90 ± 1.90%) was lower than that of the HE prepared from the flowering aerial parts of *T. mastichina* (16.39 ± 0.43%), while the HE from the capitula of *H. stoechas* presented the lowest yield (7.13 ± 1.86%). However, as previously mentioned, the extraction procedure employed enables the recovery of a broad spectrum of compounds from the plant material that are not limited to phenolic constituents [12], which may explain the higher extraction yield observed for *T. mastichina* in comparison to *L. pedunculata*.

Additionally, the consistently higher amount of phenolic compounds pointed out for *L. pedunculata* was further supported by other methodologies, particularly by the TPC assay based on GAE, where the highest results were observed for this HE. These observations are significant, as TPC in plant extracts has been reported to strongly and positively correlate with antioxidant activity [44]. This correlation arises from the fact that the carboxylic acid group may enhance the radical-scavenging potential of phenolic OH groups, thereby enhancing the antioxidant activity of phenolic acids [45]. The FTIR-PCA also allowed the identification of non-aromatic compounds, presumably carbohydrates and aliphatic compounds, prominently depicted in the spectral regions *j* (1175–1101 cm^−1^) and *k* (1077–1033 cm^−1^). These features were particularly more pronounced in the spectra of *H. stoechas*. Accordingly, the HE derived from *H*. stoechas exhibited weaker cell-free antioxidant potential, particularly when compared to the *L. pedunculata* HE.

The chemical complexity of plant-based extracts requires performing more than one assay of cell-free antioxidant potential [44]. Hence, thorough screening was carried out and noticeable differences between the results obtained from the DPPH and ABTS assays were observed. This may be explained by the ability of antioxidant compounds to act differently upon DPPH^•^ and ABTS^•+^ inhibition [46]. Moreover, these results are scarcely comparable to those of previous works given the differences in the used methodologies and data representation [47]. As such, DPPH^•^ inhibition by HE of *T. mastichina* is higher than that determined previously in aqueous extracts [48], while Barros et al. [49] showed that the effect of this species did not surpass that of trolox, the positive control. On the other hand, HE of *L. pedunculata* showed particularly interesting ABTS^•+^ inhibition, which aligns with previous findings on *Lavandula stoechas* subsp. *luisieri* (Rozeira) Rozeira HE [50].

The promising cell-free antioxidant results for *L. pedunculata* and *T. mastichina* must be due to their richness in rosmarinic acid and salvianolic acid derivatives, as shown by the FTIR-ATR analysis, which indicated a high abundance of phenolic compounds, and lastly detailed by HPLC-DAD-ESI/MS^n^. Rosmarinic acid is a hydroxycinnamic acid with two catechol groups that confer potent antioxidant activity [51]. Similarly, the antioxidant potential of molecules such as salvianolic acid B could arise from their phenolic hydroxyls, which provide them with the capacity to donate several hydrogen atoms, as already discussed for such molecules [45]. Previous studies have already reported rosmarinic acid and salvianolic acid B as major compounds in *L. pedunculata* [5,24,25,52]. Interestingly, medioresionol, a plant lignan found in a variety of plant foods was also identified [53]. To the best of our knowledge, this is the first study reporting the presence of this compound in *L. pedunculata*. Regarding phenolic acid derivatives, they were also previously identified in aqueous and hydroalcoholic extracts of *T. mastichina* [29,30,54]. However, in these previous studies, salvianolic acid A was reported to be present, which was not observed in our extracts. Instead, the B/E and K variants of salvianolic acid were detected. Overall, these studies highlight that the phenolic composition of HE of *L. pedunculata* and *T. mastichina* growing in Portuguese territories is highly conserved, with only subtle differences in their profiles of phenolic compounds. As for *H. stoechas*, this is the first time that characterization of the HE obtained from its capitula has been conducted. The major phenolic compounds identified were caffeic acid derivatives, like 4-*O*-caffeoylquinic acid and 3,5-*O*-diCaffeoylquinic acid, which is in alignment with previous reports in which other types of extractions were used for this species [20,21], and for *Helichrysum stoechas* subsp. *barrelieri* (Ten.) Nyman [22]. Furthermore, several glycosylated forms of quercetagetin were described in this *Helichrysum* species, following what has been reported for this genus [22,23]. However, differences were denoted when comparing them with Italian capitula, where kaempferol-3-*O*-glucoside was identified as a major polyphenol [20], or with the extract studied by Les et al. [23], who identified arzanol, santinol, and helipyrone. All these compounds were not detected in our extract.

Considering the detailed chemical profile established through FTIR-ATR and HPLC-DAD-ESI/MS^n^, as well as the demonstrated cell-free antioxidant potential, these phenolic-rich extracts hold promise for future skin applications. To date, the effects of the HEs from these plant species on normal skin cells have not been previously reported in the literature. For some tested concentrations, these samples revealed a significant impact on metabolic activity, rather than on cell mass, with *L. pedunculata* evoking the greatest toxic effect, particularly on NHDF cells. In comparison, *T. mastichina* showed greater biocompatibility in the studied cell lines. Possibly, the higher amounts of phenolics derivatives found in *L. pedunculata* in comparison to *T. mastichina* may justify its cytotoxic effects. Notably, their biocompatibility at non-toxic concentrations (Lp 0.2 mg/mL and Tm 0.4 mg/mL) was further ensured by proving the absence of skin irritant effects (Figure 8), in compliance with OECD Test Guidelines [33]. In fact, the absence of irritant effects has also been observed for other phenolic-rich extracts, particularly *Eucalyptus globulus* Labill., where a 3D model of reconstructed human epidermis was used [3]. This thus reinforces the potential of using phenolic-rich extracts for human skin applications.

Increased oxidative stress is a key factor that promotes cellular alterations, including DNA, lipid, and protein damage, that may lead to premature aging and even cell death by apoptosis or necrosis. From this perspective, the in vitro treatment with oxidative stressors, like *t*-BHP and H_2_O_2_, is a suitable approach to assessing the potential antioxidant protection of bioactive compounds [7,41]. From our results, *T. mastichina* stood out due to its cytoprotective capacity and as a cellular oxidative modulator in NHDF cells. Although other compounds may play a crucial role, the major identified components, rosmarinic and salvianolic acid derivatives, are likely to be responsible for the identified pharmacological activities. While salvianolic acid B has been thoroughly studied [55,56,57,58,59], the salvianolic acid K form, which is abundant in the HE from *T. mastichina* and a precursor of the B form, has not been explored. Nonetheless, it is conceivable that its properties may align with what is known about the B form, which exhibits anti-inflammatory, anti-apoptotic, and anti-fibrotic properties while also promoting the proliferation and differentiation of stem cells, in addition to having beneficial effects for the cardiac and nervous systems, as well as in aging scenarios [59]. Furthermore, the presence of free luteolin, apigenin, and naringenin aglycones, which typically exhibit higher antioxidant potential, may also promote better cytoprotective and antioxidant activities, as observed for the *T. mastichina* HE. This is differs from *L. pedunculata* and *H. stoechas* HEs, where only glycosidic derivatives were found. This is particularly interesting since it has been reported that phenolic aglycones, frequently linked to sugar moieties, may hinder the potential antioxidant role of phenolic cores to different degrees, and ultimately reduce their antioxidant activity [32,60]. As for rosmarinic acid, it showed cytoprotective effect in several cell lines, mediated by its antioxidant and caspase-3-blocking activities [61]. In fact, Hahn et al. [62] were the first to highlight the cytoprotective potential of rosmarinic acid in H_2_O_2_-induced NHDF, by reducing senescence of cells, re-activating the Nrf2-associated antioxidant defense pathway, and suppressing several pro-inflammatory factors. Interestingly, this phenolic acid also effectively protected skin fibroblasts against parabens and benzophenone-3-induced disturbances [63,64], which are common ingredients in cosmetic/sunscreen formulations. Another indicator of the cytoprotective role of rosmarinic acid came from the hydroethanolic extract (50:50%, *v*/*v*) of *Thymus vulgaris* L. (Lamiaceae), which was shown to ameliorate UVB-induced damage in NHDF cells by downregulating the mitogen-activated protein kinase/activated protein-1 (MAPK/AP-1) cascade while activating the nuclear factor erythroid 2-related factor 2-antioxidant response element (Nrf2-ARE) pathways [65]. Recently, another hydroethanolic extract (50:50%, *v*/*v*) rich in rosmarinic acid, obtained from *Salvia rosmarinus* Spenn. (Lamiaceae), was demonstrated to protect and induce elastic fiber formation in NHDF cells [66]. Similar pharmacological properties have been depicted for salvianolic acid B. This was isolated from an extract of *Salvia miltiorrhiza* Bunge (Lamiaceae), and it was demonstrated to inhibit the MAPK/AP-1 pathway in UVB-injured NHDF cells [58]. Furthermore, salvianolic acid B was also shown to effectively counteract photoaging in UVB-exposed skin fibroblasts, ultimately protecting mitochondria from excessive ROS production by enhancing Nrf2 nuclear translocation and activating antioxidant mechanisms [67].

The potential anti-inflammatory effect of these samples was also explored using a well-established LPS-induced model of inflammation in RAW 264.7 macrophages. LPS binds to toll-like receptor 4 (TLR4) at the macrophage surface, triggering nuclear factor kappa-light-chain-enhancer of activated B cells (NF-κB) translocation into the nucleus. NF-κB further promotes the transcription of a plethora of target genes, including Nos2, which encodes nitric oxide synthase (iNOS), an enzyme responsible for producing the pro-inflammatory mediator nitric oxide (NO) [3,68]. As such, the remarkable ability of *L. pedunculata* and *T. mastichina* HEs to inhibit NO production was demonstrated. On the other hand, and similarly to the absence of cytoprotective and antioxidant potential, *H. stoechas* did not present an anti-inflammatory effect. Hydroethanolic extract of *Prunella vulagris* L. (Lamiaceae), rich in rosmarinic acid, was also shown to suppress iNOS expression and decrease NO production in RAW 264.7 cells [68]. An analogous mechanism was reported for rosmarinic acid-rich extracts obtained from *Glechoma hederacea* L. (Lamiaceae) and with aqueous extracts of *Thymbra capitata* (L.) Cav. (Lamiaceae) [60]. We must remark that these authors also showed *T. capitata* extracts to be mainly composed of salvianolic acid derivatives and rosmarinic acid. Overall, these studies allow us to infer that the cytoprotective, antioxidant, and ROS modulation activities and the inhibition of cellular production of nitrites are likely driven by the chemical profile of these HEs herein studied.

As previously mentioned, excessive ROS production triggers a cascade of intracellular pathways that, in addition to causing oxidative stress, inflammation, and senescence, also contributes to skin loosening and wrinkle formation [57]. Alongside this, recent findings on the role of the non-neuronal cholinergic system in regulating skin physiology and homeostasis have highlighted the significance of acetylcholinesterase for the skin [69], in addition to the pivotal role of widely recognized skin aging-related enzymes like hyaluronidase, tyrosinase, and elastase. From this perspective, at non-toxic concentrations, *L. pedunculata* and *T. mastichina* stood out for their hyaluronidase-inhibitory activity. Similarly, *Thunbergia laurifolia* Lindl. (Acanthaceae) leaf, whose extracts are also rich in rosmarinic acid, exhibited potent hyaluronidase inhibition [70]. Yet interesting results were also found for the inhibition of elastase and tyrosinase. Also, rosmarinic acid has been proposed as a promising inhibitor of acetylcholinesterase [69,71], and only *T. mastichina* exhibited minimal potential against it. Given these cell-free anti-aging results, we believe that *T. mastichina* is a promising source of ingredients to be included in cosmetic formulations to prevent the hallmarks of skin aging.

## 4. Materials and Methods

### 4.1. Reagents and Chemicals

Detailed information about the used products is found in the Supplementary Materials.

### 4.2. Plant Material

Selected plants were harvested in Côa Valley, a territory of Northeastern Portugal. The botanical identification was confirmed by Dr Rosa Pinho from the University of Aveiro, and voucher specimens were deposited at the Herbarium of the same University (AVE). After harvesting, the plants were air-dried at room temperature (22–25 °C) and kept in the dark until extract preparation. Detailed information about plant harvesting is presented in Table S1 (Supplementary Materials). Plant names were checked according to [72] (last accessed on 14 February 2025).

### 4.3. Hydroethanolic Extract Preparation

The HEs were obtained according to the procedures previously described by our team [12]. Briefly, the dried and powdered plant material (5 g) was subjected to hydroalcoholic extraction using 125 mL of a mixture of ethanol and water (80:20, *v*/*v*, 25 °C), with magnetic stirring for 1 h. After that, the mixture was decanted, and the same plant residue was re-extracted under the same previous conditions. The two extraction solutions were pooled, filtered under vacuum conditions with a Büchner funnel, and concentrated using a vacuum rotator, and the obtained aqueous residues were subsequently freeze-dried. Further information about plant harvesting, extract preparation, and yield of extraction is presented in Table S1 (Supplementary Materials).

### 4.4. FTIR-ATR Spectroscopy Analysis

Fourier transform mid-infrared (FTIR) spectra were acquired in attenuated total reflectance (ATR) mode for the obtained freeze-dried extracts, as well as for standard phenolic compounds (Figure S1; Supplementary Materials), in the 3700–400 cm^−1^ range, following the approach previously described by our team [73,74]. Briefly, replicated representative spectra (≥20 replicate spectra per sample) were acquired using a Bruker Optics Vertex 70 FTIR spectrometer (Bruker, Billerica, MA, USA) purged with CO_2_-free dry air and equipped with a Bruker Platinum ATR single-reflection diamond accessory. A Ge-on-KBr substrate beam splitter and a liquid-nitrogen-cooled wide-band mercury cadmium telluride (MCT) detector were used. Spectra were averaged over 32 scans at a resolution of 2 cm^−1^, and the 3-term Blackman-Harris apodization function was applied. The Bruker Opus 8.1 software was used to (i) remove H_2_O and CO_2_ contributions and (ii) correct for the frequency dependence of the electric field penetration depth in ATR (considering a mean refractive index of 1.25). Absorbance spectra were converted to text files and combined into a matrix of the full dataset in MatLab (v. R2023b; MathWorks, Natick, MA, USA), where the Eigenvector PLS Toolbox (v. 9.0; Eigenvector Research, Wenatchee, WA, USA) was employed to pre-process and perform multivariate analysis of the data. Firstly, the spectral baseline was corrected using the automatic weighted least squares method (polynomial order = 2). Subsequently, the spectra were normalized, using the 2-Norm method, to normalize the rows of the matrix to unit vectors. The underlying chemometric relationships between FTIR-ATR spectra were investigated in the region of 1800–800 cm^−1^ through multivariate analysis, using the Eigenvector PLS Toolbox (v. 9.0; Eigenvector Research, Wenatchee, WA, USA).

### 4.5. HPLC–DAD–ESI/MS^n^ Analysis

The analysis of extracts was carried out according to the original method described in [75]. Accordingly, extracts were filtered through a 0.22 µm disposable liquid chromatography (LC) filter disk and were analyzed using a Dionex Ultimate 3000 UPLC (Thermo Scientific, San Jose, CA, USA) system equipped with a diode array detector coupled with an electrospray ionization mass detector (HPLC-DAD-ESI/MS^n^). Chromatographic separation was achieved with a Waters Spherisorb S3 ODS-2 C18 (3 μm, 4.6 × 150 mm, Waters, Milford, MA, USA), column-thermostatized at 35 °C. The solvents used were (A) 0.1% formic acid in water and (B) acetonitrile. The elution gradient established was isocratic 15% B (5 min), 15–20% B (5 min), 20–25% B (10 min), 25–35% B (10 min), 35–50% B (10 min), and re-equilibration of the column, using a flow rate of 0.5 mL/min. Double online detection was carried out in the DAD (using 280 and 370 nm as preferred wavelengths) and in a mass spectrometer (MS) connected to a HPLC system via the DAD cell outlet. MS analyses were conducted in negative ion mode on an LTQ XL MS Linear Ion Trap (ThermoFinnigan, San Jose, CA, USA) equipped with an ESI source. Nitrogen was used as the sheath gas (50 psi). The instrument operated with a spray voltage of 5 kV, a source temperature of 325 °C, and a capillary voltage of −20 V. The tube lens was maintained at −66 V. Full-scan spectra were acquired over an *m*/*z* range of 100–1500. Collision energy was set to 35 arbitrary units. Data acquisition was carried out with the Xcalibur^®^ data system (Thermo Finnigan, San Jose, CA, USA). The phenolic compounds were identified by comparing their retention times and their UV–Vis and mass spectra with those obtained from standard compounds, when available. Otherwise, compounds were tentatively identified by comparing the obtained information with available data reported in the literature. The quantification of the compounds was performed using 7-level calibration curves, which are summarized in the Supplementary Information. For compounds without a standard compound, the calibration curve of the most structurally similar compound was used. Results were expressed as mg of compound per g of extract.

### 4.6. Major Phenolics Estimation and Cell-Free Antioxidant Activity Assessment

The following assays were performed as stated in [76].

#### 4.6.1. Total Phenolic Content (TPC) and Total Flavonoid Content (TFC)

To determine TPC, the Folin–Ciocalteu method was carried out [76]. Briefly, 50 µL of Folin–Ciocalteu (50%, *v*/*v*) reagent and 400 µL of Na_2_CO_3_ (5%, *w*/*v*) were added to 50 µL of each test-sample, in triplicate, in a 48-well plate. The plate was shaken and incubated at room temperature for 20 min in the dark, and the absorbance was measured at 760 nm using a µQuant™ Microplate spectrophotometer (BioTek Instruments Inc., Bad Friedrichshall, Germany), against a blank consisting of a mixture of all the used solvents in equivalent amounts. In addition, gallic acid (GA) was used as a reference standard, and the TPC was expressed as mean ± SD of three independent experiments as gallic acid equivalents (GAE) per gram of extract dry weight (DW) (mg GAE g^−1^ extract DW).

The TFC of the extracts was determined by the aluminum chloride (AlCl_3_) method [76]. Therefore, 100 µL of a 1% (*w*/*v*) aluminum chloride solution was added to 100 µL of each test-sample, in triplicate, in a 96-well plate. The samples were shaken and incubated for 10 min, in the dark, at room temperature, and the absorbance was then measured at 425 nm using a µQuant™ Microplate spectrophotometer (BioTek Instruments Inc., Bad Friedrichshall, Germany) against a blank containing a mixture of all the used solvents in equivalent amounts. Quercetin (Q) was used as a reference standard, and the TFC was expressed as mean ± SD of three independent experiments as quercetin equivalents (QE) per gram of extract dry weight (DW) (mg QE g^−1^ extract DW).

#### 4.6.2. Ferric Reducing Antioxidant Power (FRAP) Assay and Cupric Ion Reducing Antioxidant Capacity (CUPRAC) Assay

The FRAP and CUPRAC assays were performed as previously described [76]. The FRAP reagent was prepared by mixing acetate buffer (0.3 M, pH = 3.6), 2,4,6-tris(2-pyridyl)-s-triazine (TPTZ, 10 mM) in 40 mM HCl, and FeCl_3_·6(H_2_O) (20 mM) in a ratio of 10:1:1 (*v*/*v*/*v*) and incubating at 37 °C for 10 min before use. Then, 10 µL of each extract concentration was reacted with 200 µL of FRAP reagent, in triplicate, in a 96-well plate. Following room-temperature incubation for 30 min, in the dark, absorbance was read at 593 nm using a µQuant™ Microplate spectrophotometer (BioTek Instruments Inc., Bad Friedrichshall, Germany) against a blank containing a mixture of all the used solvents in equivalent amounts. Trolox (T) was used as a reference standard. The FRAP activity was expressed as mean ± SD of three independent experiments as Trolox equivalents (TE) per gram of extract dry weight (DW) (mg TE g^−1^ extract DW).

A mixture of ammonium acetate buffer (1 M, pH = 7), neocuproine (7.5 mM), and CuCl_2_ (10 mM) was prepared in a ratio of 1:1:1 (*v*/*v*/*v*). Using a 96-well plate, 150 µL of this mixture was reacted with 25 µL of each extract concentration in triplicate. After incubating at room temperature for 30 min (in the dark), absorbance was read at 450 nm against a blank consisting of a reagent mixture with Milli-Q water, and Trolox (T) was used as a reference standard. The CUPRAC activity (mg TE g^−1^ extract DW) was determined as for the FRAP assay.

#### 4.6.3. 2,2-Diphenyl-1-Picrylhydrazyl (DPPH^•^) Radical and 2,2′-Azino-bis(3-ethylbenzothiazoline-6-sulfonic Acid) Radical Cation (ABTS^•+^) Scavenging Activity Assays

The DPPH^•^ and ABTS^•+^ assays were performed as previously described [76]. Firstly, the DPPH radical was prepared in methanol to obtain a maximum absorption value at 515 nm in the 0.9–1.0 range. In a 96-well plate, 100 µL of DPPH^•^ solution was added to 100 µL of different extract concentrations, in triplicate, and incubated for 30 min, at room temperature, in the dark. The DPPH^•^ scavenging was then read at 515 nm, using a µQuant™ Microplate spectrophotometer (BioTek Instruments Inc., Bad Friedrichshall, Germany) against a blank containing a mixture of all the used solvents in equivalent amounts. A mixture of DPPH^•^ and Milli-Q water (extracts solvent) was used as a negative control, and butylated hydroxytoluene (BHT) as a reference antioxidant. Using a nonlinear regression analysis, in sigmoidal dose–response curves (variable slope), the DPPH^•^ scavenging activity was determined as the half-maximal inhibitory concentration (IC_50_, mg/mL).

The ABTS radical cation was formed by adding 5 mL of a 4.9 mM potassium persulfate solution to 5 mL of a 14 mM ABTS solution and kept for 16 h in the dark. This solution was then diluted in ethanol (1:50) to obtain a maximum absorption value of 0.7 nm at 734 nm. Then, 190 µL of ABTS^•+^ radical was added to 10 µL of different extract concentrations in triplicate, in a 96-well plate. After 6 min of incubation at room temperature (in the dark), absorbance was read at 734 nm using a µQuant™ Microplate spectrophotometer (BioTek Instruments Inc., Bad Friedrichshall, Germany) against a blank containing a mixture of all the used solvents in equivalent amounts. A mixture of ABTS radical cation and Milli-Q water (extracts solvent) was used as negative control, and BHT as a reference antioxidant. The percentage of ABTS^•+^ scavenging and subsequent IC_50_ values (mg/mL) were calculated as for the DPPH assay.

### 4.7. Cell Culture

Normal human dermal fibroblasts (NHDF) cells were purchased from Lonza Group AG (Basel, Switzerland). NHDF cells were cultured in Dulbecco’s modified Eagle’s medium (DMEM) (pH 7.3), supplemented with 5 mM glucose, 1 mM sodium pyruvate, 4 mM L-glutamine, 21 mM sodium bicarbonate, 10% (*v*/*v*) of heat-inactivated fetal bovine serum (FBS), and 1% (*v*/*v*) of penicillin-streptomycin solution. The human keratinocytes (HaCaT, CLS 300493, Eppelheim, Germany) were cultured in DMEM low glucose (pH 7.3), supplemented with 35.9 mM sodium bicarbonate, 25 mM glucose, 10% (*v*/*v*) of heat-inactivated FBS, and 1% (*v*/*v*) penicillin-streptomycin solution. The mouse leukemic macrophage cell line (RAW 264.7, ATCC TIB-71, Manassas, VA, USA) was cultured in DMEM low glucose (pH 7.3), supplemented with 10% (*v*/*v*) non-inactivated FBS, 1% (*v*/*v*) penicillin-streptomycin solution, 17.95 mM sodium bicarbonate, and 25 mM glucose. Cells were detached with trypsin (NHDF and HaCaT) or using a cell scraper (RAW 264.7) to be subcultured when they reached 80–90% confluence. All cell lines were maintained at 37 °C in a humidified atmosphere of 5% CO_2_.

### 4.8. Cell Metabolic Activity

The effect of HEs on the metabolic activity of normal skin cells was evaluated following the resazurin reduction principle [77]. Briefly, NHDF and HaCaT cells were seeded at 1 × 10^4^ cells/well and 2 × 10^4^ cells/well, respectively, in a 96-well plate and left to stabilize for 24 h. After cells’ treatment with HEs (0.2–1.0 mg/mL) for 24 h more, the cells’ supernatant was discarded, and 100 µL of a solution of resazurin salt dye prepared in cell culture medium (10 µg/mL) was added to the cells. The reduction of blue-colored resazurin solution to pink-colored resorufin by metabolically active cells was determined by measuring the absorbance at 570 and 600 nm in a BioTek reader (BioTek Instruments, Inc., Winooski, VT, USA). Four independent experiments, with three experimental replicates per condition, were performed. Metabolic activity was calculated and expressed as % of the control according to Equation (1):

(1) $$Metabolic\;activity\left(\%\right)=\;\frac{\left(Abs\;570-Abs\;600\right)\;of\;SPL}{\left(Abs\;570-Abs\;600\right)\;of\;CTRL}\times 100$$

where *Abs* is absorbance in nm, *CTRL* is the control (untreated cells), and *SPL* is the samples (treated cells).

### 4.9. Cell Mass

A Sulforhodamine B (SRB) assay was performed for cell mass determination based on the measurement of cellular protein content [77,78]. The absorbance was measured at 510 nm and the background measurement at 620 nm, at room temperature, using a BioTek reader (BioTek Instruments, Inc., Winooski, VT, USA). Four independent experiments, with three experimental replicates per condition, were performed. Cell mass was calculated and expressed as % of the control following Equation (2):

(2) $$Cell\;mass\left(\%\right)=\;\frac{(Abs\;510-Abs\;620)\;of\;SPL}{(Abs\;510-Abs\;620)\;of\;CTRL}\;\times 100$$

where *Abs* is absorbance in nm, *CTRL* is the control (untreated cells), and *SPL* is the samples (treated cells).

### 4.10. Cell-Free Enzymatic Inhibition Assays

For the following assays, absorbance measurements took place in a multi-well spectrophotometer (µQuant™ Microplate spectrophotometer, BioTek Instruments Inc., Bad Friedrichshall, Germany). Five independent experiments were performed in triplicate.

#### 4.10.1. Anti-Hyaluronidase Assay

The anti-hyaluronidase effect of HEs was tested as stated in [79]. N-acetyl-D-glucosamine, arising from hyaluronic acid breakdown, reacted with dimethyl benzaldehyde in acidic conditions, and absorbance from the pink-colored chromogenic complex was recorded at 585 nm at 25 °C. EGCG (200 µM) was used as a positive control, and MilliQ water as a negative control. The blanks consisted of a mixture of the reagent’s solvents. The results are expressed as a percentage (%) of inhibition.

#### 4.10.2. Anti-Elastase Assay

The anti-elastase activity of HEs was evaluated as previously described in [69]. The formation of the product *p*-nitroaniline derived from N-succinyl-Ala-Ala-Ala-*p*-nitroanilide hydrolysis was monitored at 405 nm, every 30 s for 3 min, at 25 °C. EGCG (250 µM) was used as a positive control, and MilliQ water as the negative control. The blanks consisted of a mixture of the reagent’s solvents. The results wareere expressed as a percentage (%) of inhibition.

#### 4.10.3. Anti-Tyrosinase Assay

The anti-tyrosinase activity of HEs was evaluated as previously described in [69]. The production of dopachrome was detected from absorbance recording at 450 nm every 2 min, for 10 min, at 30 °C. Kojic acid (0.8 mM) was used as the positive control, and MilliQ water as the negative control. The blanks consisted of a mixture of the reagent’s solvents. Results were expressed as a percentage (%) of inhibition.

#### 4.10.4. Anti-Acetylcholinesterase Assay

The anti-acetylcholinesterase effect of HEs was determined following the procedures described in [71]. The absorbance was measured at 405 nm, immediately after starting the reaction, and then every 30 s for 3 min, at 25 °C. Tacrine (3 μM) was used as the positive control, and MilliQ water as the negative control. The blanks consisted of a mixture of the reagent’s solvents. The results are expressed as a percentage (%) of inhibition.

### 4.11. Measurement of Cellular Production of Nitrites

The procedure previously published in [3] was carried out to determine the production of nitrites in RAW 264.7 cells, seeded in 96-wells plate at a density of 5 × 10^4^ cells/well, and treated with HEs in the absence or presence of 0.1 µg/mL lipopolysaccharide (LPS), for 24 h. Untreated cells were used as a control (CTRL). Briefly, 100 µL of cell supernatants were reacted with 100 µL of Griess reagent in the dark at RT, for 30 min, and the absorbance was determined at 550 nm at RT, using a BioTek reader (BioTek Instruments, Inc., Winooski, VT, USA), using the culture medium as a blank. Four independent experiments, with three experimental replicates per condition, were performed, and the results are expressed as µM of nitrites produced.

### 4.12. Evaluation of Cytoprotective Efficiency

The cytoprotective effect of HE in the presence of the oxidative stressors *tert*-butyl hydroperoxide (*t*-BHP; 0.5 mM) and hydrogen peroxide (H_2_O_2_; 1.5 mM) was evaluated in NHDF as stated in [41]. Cells were seeded at 1 × 10^4^ cells/well in a 96-well plate and allowed to proliferate for 24 h before HE treatment with non-toxic concentrations of HE for another 24 h period. Finally, the extracts were removed, and the oxidative-stress-inducing agents were added to cells for 3 h more. Cellular metabolic activity and cell mass were determined as described above.

### 4.13. Determination of Intracellular Oxidative Stress

To evaluate intracellular oxidative stress, the procedure for the oxidation of 2′,7′-Dichlorodihydrofluorescein diacetate (H_2_DCFDA) fluorescent dye was followed [80]. NHDF cells were seeded in 96-well black plates with optical bottoms at 1 × 10^4^ cells/well and allowed to proliferate. After a 24 h period, HEs were added to cells for 24 h more. Afterwards, H_2_DCFDA (5 μM) was added for 2 h in medium without sodium bicarbonate nor FBS. After the 2 h period, the medium was replaced with fresh medium without the probe, sodium bicarbonate, or FBS, but with *t*-BHP at 0.5 mM, and the kinetics of the oxidizing H_2_DCFDA was immediately monitored every 5 min for 30 min using a BioTek reader (BioTek Instruments, Inc., Winooski, VT, USA) with excitation and emission wavelengths of 485 and 528 nm. Six independent experiments, with three experimental replicates per condition, were performed.

### 4.14. Skin Irritation

Skin irritation was evaluated as strictly stated in the standard operating procedure, using the SkinEthic™ Reconstructed Human Epidermis (RHE) model (EPISKIN Laboratories, Lyon, France), in compliance with OECD guidelines [33]. The tissue viability was assessed through the MTT assay. The absorbance of the formazan crystals was measured at 570 nm using a BioTek reader (BioTek Instruments, Inc., Winooski, VT, USA). Tissue viability results are expressed as % of the control. Testing samples are considered irritant if they reduce tissue viability to <50%.

### 4.15. Statistical Analysis

The results are presented as the mean ± standard deviation (SD) of the indicated number of independent experiments. The normality of the data distribution was assessed by the D’Agostino and Pearson and Shapiro–Wilk normality tests. All calculations for the descriptive statistics and one-way analysis of variance (ANOVA), as well as Tukey’s, Dunnett’s, and Sidak’s range tests, were performed in GraphPad Prism 9.0 software (GraphPad Software, La Jolla, CA, USA), considering the following significance values: * *p* < 0.05, ** *p* < 0.01, *** *p* < 0.001, and **** *p* < 0.0001, as well as ^#^ *p* < 0.05, ^##^ *p* < 0.01, ^###^ *p* < 0.001, and ^####^ *p* < 0.0001.

## 5. Conclusions

This study unveiled the potential of phenolic-rich extracts from *H. stoechas*, *L. pedunculata*, and *T. mastichina*, which have long been used in traditional medicine in Mediterranean regions. To support the valorization of these bioactive ingredients, their chemical profiles were comprehensively characterized, and non-toxic concentrations of their HEs were determined through a robust screening assay. Subsequently, key pharmacological properties relevant to skin aging were explored. From these studies, the *T. mastichina* HE, rich in phenolic acid derivatives such as rosmarinic and salvianolic acids, stood out as the most promising given its antioxidant, cytoprotective, and potential anti-inflammatory activities, alongside its relevant anti-hyaluronidase effect. These observations reinforce the importance of conducting in-depth studies, supporting our aim of elucidating its mechanisms of action as an anti-inflammatory and antioxidant agent, two pivotal factors regarding skin aging. Alongside this, our findings further demonstrated that the FTIR-ATR technique, when combined with multivariate analysis (PCA), is a feasible approach for the direct examination of plant extracts, enabling the identification of their chemical characteristics and the relative abundance of their principal components, acting as a driving force for subsequent assays, which, in this case, enabled the exploration of these plants as rich sources of ingredients to manage human skin aging effects. Ultimately, our findings align with the increasing demand for sustainable, plant-based solutions in cosmetics and pharmaceuticals, reinforcing the relevance of this research in developing products that combine toxicological safety and efficacy with environmental responsibility.

## Figures and Tables

**Table 2:** Peaks, retention times (Rt) in min, wavelengths (λ_max_) in nm, mass spectral data ([M-H]^−^ and MS^n^ (*m*/*z*)), tentative identification, and quantification (mg g^−1^ extract) of phenolic compounds in the HE of *H. stoechas*, *L. pedunculata*, and *T. mastichina*. Major compounds identified are highlighted in bold.

***H. stoechas***						
**Peak**	**Rt**	***λ*_max_**	**[M-H]^−^**	**MS^n^**	**Tentative Identification**	**Quantification**
1*^Hs^*	4.80	325	353	MS^2^: 191(100); MS^3^: 179(35), 173(>5), 161(5), 135(7)	1-*O*-Caffeoylquinic acid	1.52 ± 0.05
**2*^Hs^***	**7.28**	**326**	**353**	**MS^2^: 191(100);** **MS^3^: 179(>5), 173(135), 111(100)**	**4-*O*-Caffeoylquinic acid**	**15.95 ± 0.93**
3*^Hs^*	8.78	319	353	MS^2^: 191(100); MS^3^: 179(>5), 173(100), 111(10)	5-*O*-Caffeoylquinic acid	0.91 ± 0.04
4*^Hs^*	10.53	320	179	MS^2^: 161(100), 135(25)	Caffeic acid	0.34 ± 0.01
5*^Hs^*	13.69	328	367	MS^2^: 191(100); MS^3^: 173(56), 127(100)	5-*O*-Feruloylquinic acid	0.52 ± 0.02
6*^Hs^*	15.02	355	479	MS^2^: 317(100)	Myricetin-3-*O*-hexoside	4.47 ± 0.02
7*^Hs^*	16.32	321	873	MS^2^: 829(44), 625(100); MS^3^: 479(100), 317(23)	Quercetagetin 3-*O*-(malonylcoumaroyl)- hexoside-7-*O*-hexoside isomer I	2.73 ± 0.01
8*^Hs^*	16.71	322	787	MS^2^: 625(100); MS^3^: 479(100), 317(23)	Quercetagetin-*O*-coumaroyl- hexosyl-*O*-hexoside	2.93 ± 0.03
9*^Hs^*	17.16	323	461	MS^2^: 317(100)	Quercetagetin or mirycetin derivative isomer I	2.86 ± 0.00
**10*^Hs^***	**17.79**	**320**	**873**	**MS^2^: 829(44), 625(100);** **MS^3^: 479(100), 317(23)**	**Quercetagetin 3-*O*-(malonylcoumaroyl)-** **hexoside-7-*O*-hexoside isomer II**	**5.35 ± 0.06**
**11*^Hs^***	**18.03**	**357**	**461**	**MS^2^: 317(100)**	**Quercetagetin or mirycetin derivative isomer II**	**10.79 ± 0.06**
**12*^Hs^***	**18.86**	**341**	**461**	**MS^2^: 317(100)**	**Quercetagetin or mirycetin derivative isomer III**	**3.57 ± 0.01**
13*^Hs^*	19.12	335	463	MS^2^: 301(100)	Quercetin-3-*O*-glucoside	2.43 ± 0.11
14*^Hs^*	20.29	347	549	MS^2^: 505(100), 301(23)	Quercetin-*O*-malonyl-hexoside isomer I	1.29 ± 0.02
**15*^Hs^***	**20.72**	**323**	**515**	**MS^2^: 353(100), 335(<5);** **MS^3^: 191(100), 179(74), 173(<5), 161(14), 135(<5)**	**3,5-*O*-diCaffeoylquinic acid**	**10.18 ± 0.64**
**16*^Hs^***	22.57	335	549	MS^2^: 505(100), 301(23)	Quercetin-*O*-malonyl-hexoside isomer II	1.49 ± 0.01
**17*^Hs^***	**23.13**	**329**	**515**	**MS^2^: 353(100), 335(26);** **MS^3^: 191(22), 179(31), 173(100)**	**3,4-*O*-diCaffeoylquinic acid**	**7.41 ± 0.02**
18*^Hs^*	23.62	341	515	MS^2^: 353(100), 335(<5); MS^3^: 191(34), 179(9), 173(100), 135(6)	4,5-*O*-diCaffeoylquinic acid	2.45 ± 0.17
19*^Hs^*	23.73	350	477	MS^2^: 315(100)	Isorhamnetin-*O*-hexoside	2.26 ± 0.01
20*^Hs^*	24.76	329	601	MS^2^: 557(100), 515(<5), 439(17), 395(763), 377(<5)	Malonyl-dicaffeoyl-quinic acid isomer	2.20 ± 0.12
21*^Hs^*	25.59	364	463	MS^2^: 301(100)	Quercetin-*O*-hexoside	2.13 ± 0.07
22*^Hs^*	26.07	343	563	MS^2^: 519(100), 315(35)	Isorhamnetin-*O*-(-*O*-malonyl)-hexoside	1.11 ± 0.01
23*^Hs^*	27.72	364	549	MS^2^: 285(100)	Luteolin-*O*-dipentoside	2.18 ± 0.06
24*^Hs^*	29.50	332	727	MS^2^: 683(100), 317(12)	Myricetin-*O*-malonyl-dihexoside	3.15 ± 0.09
25*^Hs^*	30.13	314	609	MS^2^: 463(23), 301(100)	Quercetin 3-*O*-[*p*-coumaroyl]-hexoside	2.47 ± 0.03
26*^Hs^*	32.31	315	695	MS^2^: 651(32), 100(>5), 301(34)	Quercetin-*O*-malony[*p*-coumarouyl]-deoxyhexoside	0.75 ± 0.02
27*^Hs^*	33.22	328	593	MS^2^: 285(100)	Kaempherol-*O*-deoxyhexosyl-hexoside isomer I	0.93 ± 0.01
28*^Hs^*	33.56	332	593	MS^2^: 285(100)	Kaempherol-*O*-deoxyhexosyl-hexoside isomer II	0.92 ± 0.02
29*^Hs^*	33.97	315	623	MS^2^: 477(25), 315(100)	Isorhamentin-*O*-deoxyhexosyl-hexoside	1.14 ± 0.04
30*^Hs^*	35.17	315	679	MS^2^: 635(12), 285(100)	Kaempherol-*O*-deoxyhexosyl-malonyl-hexoside	0.86 ± 0.01
					**Total phenolic compounds**	97.27 ± 2.33 ^a^
					**Total phenolic acids**	41.47 ± 1.75 ^b^
					**Total flavonoids**	55.80 ± 0.58 ^c^
***L. pedunculata***						
**Peak**	**Rt**	***λ*** **_max_**	**[M-H]^−^**	**MS^n^**	**Tentative Identification**	**Quantification**
1*^Lp^*	5.97	324	341	MS^2^: 179(100), 135(12)	Caffeic acid hexoside	0.53 ± 0.02
2*^Lp^*	6.51	322	489	MS^2^: 179(23), 161(100)	Caffeic acid derivative	0.89 ± 0.01
3*^Lp^*	7	326	325	MS^2^: 163(100)	*p*-Coumaroyl hexoside isomer I	1.66 ± 0.06
4*^Lp^*	7.25	324	325	MS^2^: 163(100)	*p*-Coumaroyl hexoside isomer II	1.16 ± 0.04
5*^Lp^*	8.74	311	387	MS^2^: 369(15), 207(100), 163(70)	Medioresinol	1.44 ± 0.11
6*^Lp^*	10.64	320	179	MS^2^: 161(100), 135(25)	Caffeic acid	1.26 ± 0.07
7*^Lp^*	11.87	324	325	MS^2^: 163(100)	*p*-Coumaroyl hexoside isomer III	2.68 ± 0.05
9*^Lp^*	15.28	320	357	MS^2^: 151(12), 177(32), 195(100)	Trihydroxycinnamic acid-*O*-hexoside	0.64 ± 0.01
**10*^Lp^***	**16.22**	**284.326sh**	**463**	**MS^2^: 287(100)**	**Eriodyctiol-*O*-hexuronoside**	**11.61 ± 0.37**
**11*^Lp^***	**17.44**	**319**	**521**	**MS^2^: 359(100);** **MS^3^: 197(25), 179(46), 161(100), 135(5)**	**Rosmarinic acid hexoside**	**6.39 ± 0.24**
**12*^Lp^***	**18.43**	**346**	**461**	**MS^2^: 285(100)**	**Luteolin-7-*O*- hexuronoside**	**12.59 ± 0.57**
13*^Lp^*	19.17	344	447	MS^2^: 285(100)	Luteolin-*O*-hexoside	3.96 ± 0.26
14*^Lp^*	19.45	285.332sh	719	MS^2^: 539(33), 521(<5), 359(100); MS^3^: 197(87), 179(100), 161(54), 135(5)	Sangerinic acid	3.99 ± 0.23
**15*^Lp^***	**22.15**	**326**	**359**	**MS^2^: 197(29), 179(34), 161(100)**	**Rosmarinic acid**	**60.8 ± 0.21**
16*^Lp^*	**22.95**	**335**	**445**	**MS^2^: 269(100)**	**Apigenin-*O*-hexuronoside**	**8.65 ± 0.45**
17*^Lp^*	23.42	334	431	MS^2^: 269(100)	Apigenin-*O*-hexoside	4.28 ± 0.24
18*^Lp^*	23.84	338	533	MS^2^: 489(100), 285(34)	Luteolin-*O*-malonyl-hexoside	1.34 ± 0.02
19*^Lp^*	24.37	344	475	MS^2^: 299(100), 284(54)	Methylluteolin-*O*-hexuronoside	4.31 ± 0.01
20*^Lp^*	24.96	339	461	MS^2^: 299(100), 284(32)	Methylluteolin-*O*-hexoside	1.66 ± 0.07
**21*^Lp^***	**25.61**	**311**	**717**	**MS^2^: 537(100);** **MS^3^: 339(34), 321(100), 313(<5), 295(<5), 197(13)**	**Salvianolic acid B**	**45.69 ± 0.46**
**22*^Lp^***	**28.29**	**331**	**473**	**MS^2^: 269(100)**	**Apigenin-*O*-acetyl-hexoside**	**7.81 ± 0.09**
23*^Lp^*	29.11	328	533	MS^2^: 489(65), 285(100)	Luetolin-*O*-diacetyl-hexoside	2.34 ± 0.02
26*^Lp^*	32.49	326	623	MS^2^: 285(00)	Luteolin-O-hexosyl-hexuronoside	0.67 ± 0.03
					**Total phenolic compounds**	186.34 ± 0.77 ^a^
					**Total phenolic acids**	127.14 ± 0.39 ^b^
					**Total flavonoids**	59.21 ± 0.38 ^c^
***T. mastichina***						
**Peak**	**Rt**	***λ*** **_max_**	**[M-H]^−^**	**MS^n^**	**Tentative Identification**	**Quantification**
1*^Tm^*	6.77	326	353	MS^2^: 191(100); MS^3^: 179(>5), 173(135), 111(100)	4-*O*-caffeoylquinic acid	1.39 ± 0.09
2*^Tm^*	8.82	255	387	MS^2^: 369(87), 207(100), 163(12)	Hydroxyjasmonic	0.27 ± 0.01
4*^Tm^*	10.06	356	593	MS^2^: 473(54), 383(38), 353(65)	Apigenin-*C*-dihexoside	0.88 ± 0.02
5*^Tm^*	10.6	323	179	MS^2^: 161(100), 135(25)	Caffeic acid	0.96 ± 0.02
6*^Tm^*	11.45	284	449	MS^2^: 287(100)	Eriodyctiol-*O*-hexoside isomer I	1.43 ± 0.01
7*^Tm^*	12.89	284	449	MS^2^: 287(100)	Eriodyctiol-*O*-hexoside isomer II	3.69 ± 0.10
**8*^Tm^***	**15.92**	**353**	**463**	**MS^2^: 301(100)**	**Quercetin-*O*-hexoside isomer I**	**15.23 ± 0.23**
9*^Tm^*	16.47	343	463	MS^2^: 301(100)	Quercetin-*O*-hexoside isomer II	3.32 ± 0.02
10*^Tm^*	16.97	342	447	MS^2^: 285(100)	Luteolin-*O*-hexoside isomer I	1.38 ± 0.09
11*^Tm^*	17.13	282/324	433	MS^2^: 271(100)	Naringenin-*O*-hexoside	2.73 ± 0.10
12*^Tm^*	17.5	319	521	MS^2^: 359(100); MS^3^: 197(25), 179(46), 161(100), 135(5)	Rosmarinic acid hexoside	1.63 ± 0.10
**14*^Tm^***	**19.15**	**350**	**447**	**MS^2^: 285(100)**	**Luteolin-*O*-hexoside isomer II**	**9.42 ± 0.38**
**15*^Tm^***	**19.29**	**343**	**447**	**MS^2^: 285(100)**	**Luteolin-*O*-hexoside isomer III**	**7.10 ± 0.49**
16*^Tm^*	21.07	334	449	MS^2^: 287(100)	Eriodyctiol-*O*-hexoside isomer III	1.41 ± 0.05
**17*^Tm^***	**21.59**	**338**	**555**	**MS^2^: 493(100), 359(23)**	**Salvianolic acid K isomer I**	**3.30 ± 0.15**
**18*^Tm^***	**22.05**	**341**	**555**	**MS^2^: 493(100), 359(23)**	**Salvianolic acid K isomer II**	**3.94 ± 0.04**
**19*^Tm^***	**22.32**	**320**	**359**	**MS^2^: 197(29), 179(34), 161(100)**	**Rosmarinic acid**	**36.64 ± 0.50**
**20*^Tm^***	**23**	**331**	**555**	**MS^2^: 493(100), 359(23)**	**Salvianolic acid K isomer III**	**3.19 ± 0.01**
**21*^Tm^***	**23.46**	**335**	**431**	**MS^2^: 269(100)**	**Apigenin-*O*-hexoside**	**6.02 ± 0.35**
**22*^Tm^***	**23.69**	**332**	**717**	**MS^2^: 555(20), 519(100), 475(12), 357(32)**	**Salvianolic acid B/E**	**8.62 ± 0.33**
23*^Tm^*	25.55	335	475	MS^2^: 299(100)	Chrysoeriol-*O*-hexuronoside	0.99 ± 0.01
24*^Tm^*	26.09	343	497	MS^2^: 299(100)	Chrysoeriol derivative	1.23 ± 0.06
25*^Tm^*	26.7	336	639	MS^2^: 301(100)	Quercetin-*O*-hexoside-hexuronoside	2.86 ± 0.09
26*^Tm^*	27.79	319	609	MS^2^: 301(100)	Quercetin-*O*-hexosyl-deoxyhexoside	1.23 ± 0.03
28*^Tm^*	29.17	330	609	MS^2^: 285(100)	Luteolin-*O*-dihexoside	1.31 ± 0.12
29*^Tm^*	29.95	331	623	MS^2^: 285(100)	Luteolin-*O*-hexoside-hexuronoside	1.72 ± 0.06
30*^Tm^*	32.58	344	285	-	Luteolin	1.76 ± 0.04
31*^Tm^*	35.1	289	271	-	Naringenin	1.52 ± 0.02
32*^Tm^*	37.51	335	269	-	Apigenin	3.42 ± 0.09
					**Total phenolic compounds**	128.60 ± 0.40 ^a^
					**Total phenolic acids**	59.95 ± 0.48 ^b^
					**Total flavonoids**	68.64 ± 0.07 ^c^

Values represent the mean ± standard deviation. The statistical analysis was carried out by one-way ANOVA, followed by Tukey’s post hoc test (*p* < 0.05), regarding major groups of compounds (e.g., total phenolic acids) identified in each HE. Significant differences are represented by superscript letters (a–c). Rt: retention time in min; λ_max_: wavelength (nm) of maximum absorption in the UV–visible region; [M-H]^−^: deprotonated ion (negative ion mode) (*m*/*z*); MS^n^ fragment ions generated in MS^2^ and/or MS^3^ spectra (*m*/*z*) with relative abundance in brackets (% base peak); sh: shoulder.

**Table 3:** Cell-free effect of HEs on the inhibition (%) of enzymes involved in skin aging.

	Hyaluronidase	Tyrosinase	Elastase	Acetylcholinesterase
Hs (0.2 mg/mL)	17.46 ± 6.88 ****	65.01 ± 7.96 ****	29.24 ± 7.15 ****	n.a.
Lp (0.2 mg/mL)	79.86 ± 6.70 *	64.15 ± 6.02 ****	n.a.	n.a.
Tm (0.4 mg/mL)	91.52 ± 4.19	62.65 ± 6.98 ****	28.85 ± 5.65 ****	27.37 ± 4.92 ****
Positive control ^a^	98.76 ± 8.20	93.31 ± 5.53	68.17 ± 5.51	93.97 ± 3.61

^a^ KA at 800 µM, EGCG at 200 µM, EGCG at 250 µM, and tacrine at 3 µM were used as positive controls for the tyrosinase, hyaluronidase, elastase, and acetylcholinesterase assays, respectively. Values are presented as % of enzyme inhibition and represent the mean ± SD of at least five independent experiments performed in triplicate. The statistical analysis was carried out by one-way ANOVA followed by Dunnett’s multiple comparisons test (* *p* < 0.05 and **** *p* < 0.0001 versus positive control). Abbreviations: n.a., not active; EGCG, Epigallocatechin gallate; KA, Kojic acid; Hs, *H. stoechas*; Tm, *T. mastichina*; and Lp, *L. pedunculata*.

## Data Availability

All data generated or analyzed during this study are included in this published article [and its Supplementary Materials], and raw data is available from the corresponding author on reasonable request.

## Supplementary Materials

The following supporting information can be downloaded at https://www.mdpi.com/article/10.3390/ph18121889/s1: List of reagents and chemicals used in the experiments; Table S1. Plant material harvested in Côa Valley (Portugal) assessed in this study. Information includes taxa (plant species and botanical family), the collector’s name, herbarium voucher specimen code that is deposited at the Herbarium of the University of Aveiro (AVE), date and detailed harvesting site, the employed extraction method, the plant parts used for extraction, and respective yield of extraction values (%) represented as the mean ± SD of three independent experiments. Table S2. Total phenolic content (TPC, mg GAE g^−1^ extract DW), total flavonoid content (TFC, mg QE g^−1^ extract DW), ferric (FRAP) and cupric (CUPRAC) reducing powers (mg TE g^−1^ extract DW), and free radical scavenging activity (DPPH and ABTS) presented as IC_50_ values (mg/mL); Figure S1. FTIR-ATR spectra in the range 1800–800 cm^−1^ of the standard phenolic compounds gallic acid (A), myricetin (B), quercetin (C), catechin (D), kaempferol (E), epicatechin (F), ferulic acid (G) and *p*-coumaric acid (H); Figure S2. Illustrative phenolic profiles of the HE of *H. stoechas* (A,B), *T. mastichina* (C,D), *L. pedunculata* (E,F) recorded at 280 and 370 nm, respectively. Abbreviations: mAU, milli-absorbance unit; Hs, *H. stoechas*; Tm, *T. mastichina*; Lp, *L. pedunculata*.; List of standard calibration curves used for quantification of the identified compounds in the HPLC-DAD-ESI-MS^n^ analysis; Figure S3. Effect of the HE of *H. stoechas* (A_1_,A_2_), *L. pedunculata* (B_1_,B_2_), *T. mastichina* (C_1_,C_2_) on the metabolic activity and cell mass of Raw 264.7 macrophages, respectively. Cells were treated with HE (0.2–1.0 mg/mL) and LPS (0.1 µg/mL) for 24 h and metabolic activity effects were evaluated by the Alamar blue^®^ and SRB assays. Untreated cells were used as control (CTRL). The results are expressed as percentage (%) of metabolic activity and cell mass relative to the CTRL and represent the mean ± SD of four independent experiments, each one performed in triplicates. The statistical analysis was carried out by one- ANOVA followed by Dunnett’s multiple comparison test (* *p* < 0.05, ** *p* < 0.01, *** *p* < 0.001, and **** *p* < 0.0001 versus CTRL); can be downloaded at (To be completed by the Editorial Office of Pharmaceuticals).

## Abbreviations

The following abbreviations are used in this manuscript:

ABTS^•+^	2,2′-azino-bis(3-ethylbenzothiazoline-6-sulfonic acid) radical cation
ANOVA	Analysis of variance
AVE	Herbarium of the University of Aveiro
BHT	Butylated hydroxytoluene
CUPRAC	Cupric ion reducing antioxidant capacity
DMEM	Dulbecco’s modified Eagle’s medium
DPPH^•^	2,2-Diphenyl-1-picrylhydrazyl radical
DW	Dry weight
EGCG	(-)Epigallocatechin gallate
FBS	Fetal bovine serum
FRAP	Ferric reducing antioxidant power
FTIR-ATR	Fourier transform infrared spectroscopy (FTIR) in attenuated total reflectance (ATR) mode
GAE	Gallic acid equivalents
HaCaT	Immortalized human keratinocytes cell line
HE	Hydroethanolic extract (80:20%, *v*/*v*) (EtOH 80%)
HPLC-DAD-ESI/MS^n^	High-performance liquid chromatography coupled with photodiode array detection and electrospray ionization tandem mass spectrometry
H_2_DCFDA	2′,7′-Dichlorodihydrofluorescein diacetate
Hs	*H. stoechas*
KA	Kojic acid
LC	Liquid chromatography
Lp	*L. pedunculata*
LPS	Lipopolysaccharide
MAPK/AP-1	Mitogen-activated protein kinase/activated protein-1
min	Minutes
MS	Mass spectrometer
MTT	Thiazolyl blue tetrazolium bromide
NF-κB	Nuclear factor kappa-light-chain-enhancer of activated B cells
NHDF	Normal human dermal fibroblast cell line
NO	Nitric oxide
Nrf2	Nuclear factor erythroid 2-related factor 2
OECD	Organization for Economic Co-operation and Development
OH	Hydroxyl group
QE	Quercetin equivalents
RHE	Reconstructed human epidermis
RNS	Reactive nitrogen species
ROS	Reactive oxygen species
RT	Room temperature
Rt	Retention time
SD	Standard deviation
Sec	Seconds
SRB	Sulforhodamine B
TE	Trolox equivalents
TFC	Total flavonoid content
Tm	*T. mastichina*
TPC	Total phenolic content
UV	Ultraviolet
