# Supplementary material for: A Spectrochemically Driven Study: Identifying Phenolic-Rich Extracts from Helichrysum stoechas, Lavandula pedunculata, and Thymus mastichina with Potential to Revert Skin Aging Effects
Source: Pharmaceuticals (Basel). 2025 Dec 14;18(12):1889. doi: 10.3390/ph18121889 (PMC12735882; doi:10.3390/ph18121889)
Supplement: Supplementary file 1 [file pharmaceuticals-18-01889-s001.zip › pharmaceuticals-4027674-supplementary.pdf]

Supplementary Material

List of reagents and chemicals used in the experiments:

From Sigma-Aldrich (St. Louis, MO, USA) we obtained sodium bicarbonate (S5761), D-(+)-Glucose (G8270), Dulbecco's Modified Eagle's Medium without glucose (DMEM5030), L-glutamine (G3126), sodium pyruvate (P5280), penicillin-streptomycin solution (P4333), 0.25% Trypsin-EDTA solution (T4049), trypan blue solution (T8154), dimethyl sulfoxide (DMSO) (D2438), 2,2-diphenyl-1-picrylhydrazyl (D9132), 2,4,6-tris(2-pyridyl)-*s*-triazine (≥99%) (93285), ammonium acetate (≥98%) (A7262), butylated hydroxytoluene (BHT) (≥99%) (W218405), gallic acid (97.5-102.5% (titration)) (G7384), (+)-Catechin hydrate (≥98%) (C125), ferulic acid (PHR1791), quercetin (≥95%) (Q4951), neocuproine (≥98%) (N1501), sodium acetate (≥99%) (S5636), trolox (97%) (238813), iron (III) chloride $FeCl_3 \cdot 6(H_2O)$, (≥98%) (31232-M), Lipopolysaccharide (LPS) from *Escherichia coli* serotype 026:B6 (L2654), acetylcholinesterase from *Electrophorus electricus* (electric eel) (C3389), acetylthiocholine iodide (≥98% (TLC)) (A5751), 5,5′-Dithio-*bis*-(2-nitrobenzoic Acid) (DTNB) (322123), *N*-(1-naphthyl)-ethylenediamine dihydrochloride (>98%) (222488), sulphanilamide (≥99%) (S9251), *tert*-Butyl hydroperoxide solution (*t*-BHP) 70 wt. % in H_2O (458139), hydrogen peroxide (H_2O_2) 30 % (w/w) in H_2O (H1009), thiazolyl blue tetrazolium bromide (MTT) (≥97.5%) (M5655), sodium dodecyl sulfate (SDS) (≥98.5%) (L4509), N-Succinyl-Ala-Ala-Ala-*p*-nitroanilide (≥98%) (S4760), tyrosinase from mushroom (T3824), L-Tyrosine (99.0-101.0%) (T8566), kojic acid (≥98.5%) (K3125), and sodium hydroxide (NaOH) (≥98%) (S5881). The reagents aluminum chloride ($AlCl_3$, for synthesis) (801081), copper (II) chloride ($CuCl_2$, for synthesis) (818247), potassium chloride (KCl) (≥99%) (104936), sodium carbonate (≥99.5%) (106392), and sodium acetate trihydrate ($CH_3COONa*3H_2O$) (99.5%) were obtained from Merck (Oeiras, Portugal). Fetal bovine serum (FBS) (S1810-500) and DMEM Low glucose (P0061-N10L) were acquired from BioWest (Nuaillé, France). Resazurin sodium salt (B21187), Sulforhodamine B sodium salt (SRB) (A14769.06), Tris Base (BP152-1), 2,2′-Azino-bis(3-ethylbenzothiazoline-6-sulphonic acid) diammoniumsalt (≥98%) (J65535.06), *p*-Coumaric acid (*trans*-4-Hydroxycinnamic acid) (≥97.5%) (A15167.14), 2',7'-dichlorodihydrofluorescein diacetate (H_2DCFDA) (D399), phosphoric acid 85% aqueous solution (A18067.0D) and potassium metaborate hydrate (KBO_2*H_2O) (99.98%) (047365.30) were purchased from Thermo Scientific Chemicals (Waltham, MA, USA). Glacial acetic acid (27225), methanol (24229), ethanol absolute (24194), hydrochloric acid (HCl) (37-38.0 %) (30721) and potassium persulfate (≥99%) (216224) were bought from Honeywell (Carnaxide, Portugal). The Folin–Ciocalteu's reagent (251567.1609) was purchased from Panreac (Barcelona, Spain). From Carlo Erba Reagents (Milan, Italy) we acquired calcium chloride dihydrate ($CaCl_2*2H_2O$) (99.0 - 105.0%) (327607). Kaempferol (≥98.0%) (7W-GP7425),

epicatechin (≥95.0%) (BP-BP0538) and myricetin (≥97.0%) (3B-M2131) were bought from Cymit Química (Pamplona, Spain), while from TargetMol it was purchased (-)-Epigallocatechin Gallate (EGCG) (99.43%) (T2988). From Cayman Chemical (Ann Arbor, Michigan, USA) we bought Tacrine (hydrochloride) (≥98%) (70240).

Table S1. Plant material harvested in Côa Valley (Portugal) assessed in this study. Information includes taxa (plant species and botanical family), the collector's name, herbarium voucher specimen code that is deposited at the Herbarium of the University of Aveiro (AVE), date and detailed harvesting site, the employed extraction method, the plant parts used for extraction, and respective yield of extraction values (%) represented as the mean ± SD of three independent experiments.

Taxa	Collector	Herbarium voucher specimen	Date of harvesting	Harvesting site	Type of extraction	Plant parts used for extraction	Extraction yield (%)
Helichrysum stoechas (L.) Moench subsp. *stoechas* (Hs) (Asteraceae)	Mário Pedro Marques	AVE313	May 2022	Côa Parque Foundation, V. N. de Foz Coa (41°04'49.7"N 7°06'41.0"W)	Ethanol-water (80:20, v/v)	Capitula	7.133 ± 1.857
Thymus mastichina (L.) L. subsp. *mastichina* (Tm) (Lamiaceae)	Mário Pedro Marques	AVE221	May 2021	Road from Muxagata to the archeologic rock art site of Ribeira de Piscos, V. N. de Foz Coa (41°01'40.1"N 7°08'46.5"W)	Ethanol-water (80:20, v/v)	Flowering aerial parts	16.394 ± 0.434
Lavandula pedunculata (Mill.) Cav. (Lp) (Lamiaceae)	Mário Pedro Marques	AVE156	May 2022	Côa Parque Foundation, V. N. de Foz Coa (41°04'49.7"N 7°06'41.0"W)	Ethanol-water (80:20, v/v)	Flowering aerial parts	10.897 ± 1.904

Table S2. Total phenolic content (TPC, mg GAE g^{-1} extract DW), total flavonoid content (TFC, mg QE g^{-1} extract DW), ferric (FRAP) and cupric (CUPRAC) reducing powers (mg TE g^{-1} extract DW), and free radical scavenging activity (DPPH and ABTS) presented as IC_{50} values (mg/mL).

Samples	TPC	TFC	CUPRAC	FRAP	DPPH	ABTS
**Hs**	119.616 ± 15.458^{a}	36.671 ± 4.556^{a}	431.868 ± 46.317^{a}	217.902 ± 1.676^{a}	0.08 ± 0.001^{a}	0.518 ± 0.007^{a}
**Tm**	182.463 ± 13.543^{b}	84.176 ± 5.149^{b}	480.711 ± 51.527^{b}	237.456 ± 2.283^{a}	0.071 ± 0.003^{a,b}	0.505 ± 0.03^{a,b}

**Lp**	232.823 ± 28.76[b]	33.114 ± 3.258[a]	702.593 ± 52.427[c]	408.994 ± 7.899[b]	0.049 ± 0.004[b]	0.276 ± 0.014[c]
**BHT**	-	-	-	-	0.123 ± 0.018[c]	0.164 ± 0.025[d]

Abbreviations: Hs, *H. stoechas*; Tm, *T. mastichina*; Lp, *L. pedunculata*; BHT, butylated hydroxytoluene (positive control); DW, Dry weight; GAE, gallic acid equivalents; n.d., not determined; QE, quercetin equivalents. Values represent the mean ± SD of three independent experiments performed in triplicates. For each column, different superscript letters (a-d) indicate significant differences. The statistical analysis was carried out by one-way analysis of variance (ANOVA), followed by Tukey's post hoc test ($p < 0.05$) for comparisons between three or more groups.

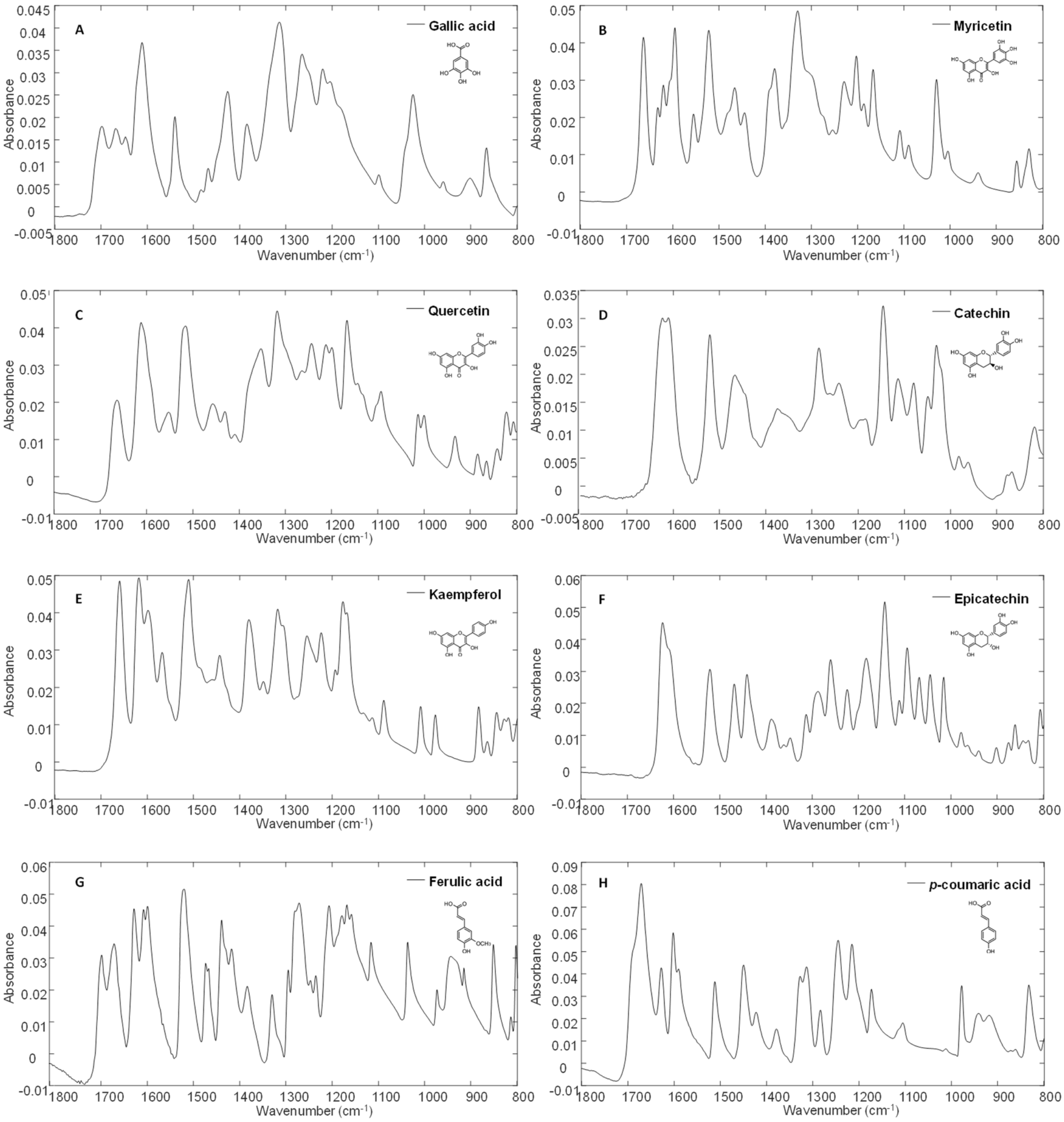

Figure S1. FTIR-ATR spectra in the range 1800 – 800 cm^{-1} of the standard phenolic compounds gallic acid (A), myricetin (B), quercetin (C), catechin (D), kaempferol (E), epicatechin (F), ferulic acid (G) and *p*-coumaric acid (H).

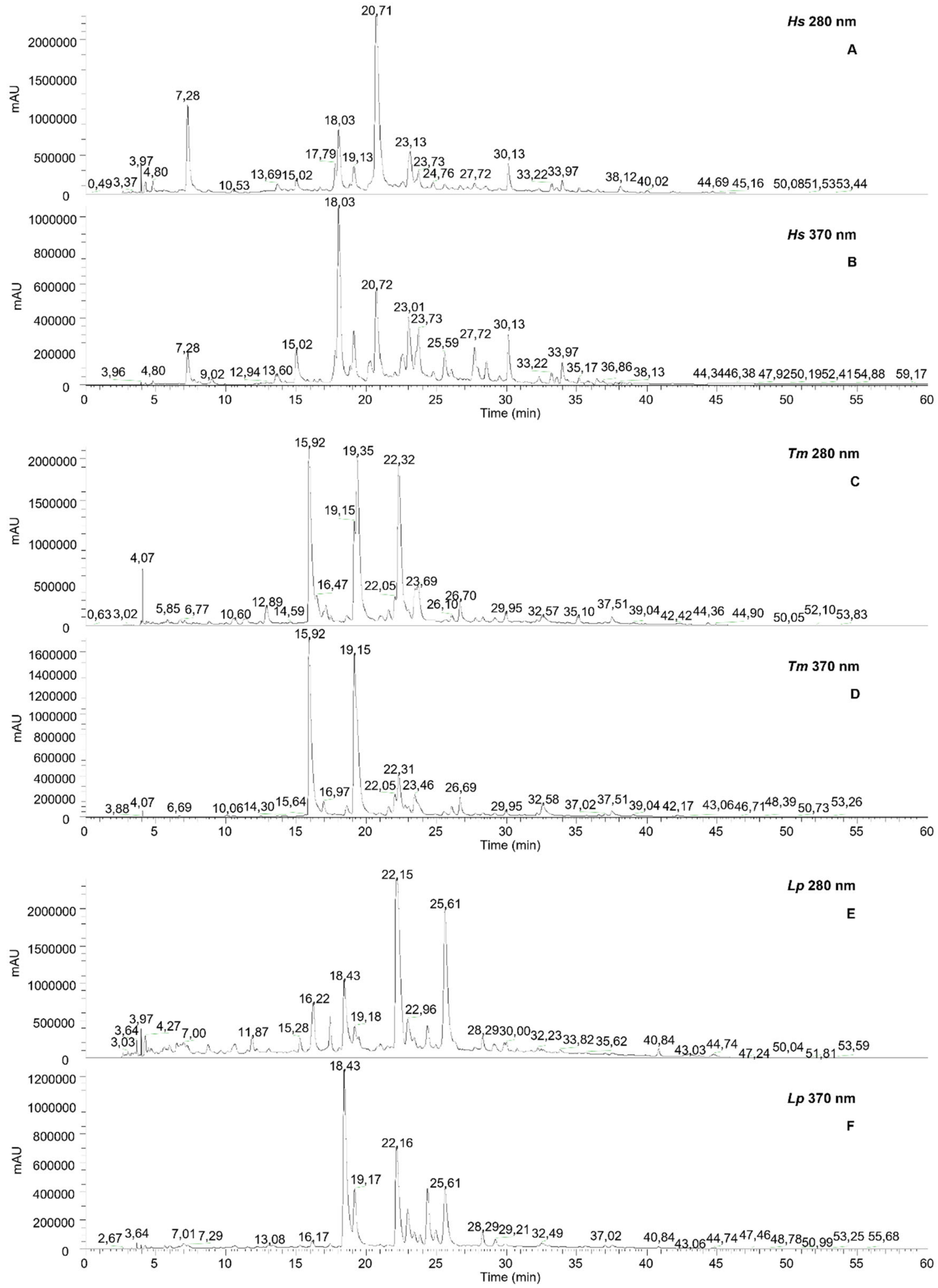

Figure S2. Illustrative phenolic profiles of the HE of *H. stoechas* (A and B), *T. mastichina* (C and D), *L. pedunculata* (E and F) recorded at 280 and 370 nm, respectively. Abbreviations: mAU, milli-absorbance unit; Hs, *H. stoechas*; Tm, *T. mastichina*; Lp, *L. pedunculata*.

List of standard calibration curves used for quantification of the identified compounds in:

***H. stoechas* (*Hs*):** Chlorogenic acid (y = 168.823x – 161.172, R^2 = 0.999; LOD = 0.20 μg/mL; LOQ = 0.68 μg/mL, peaks 1^{Hs}, 2^{Hs}, 3^{Hs}, 15^{Hs}, 17^{Hs}, 18^{Hs} and 20^{Hs}), caffeic acid (y = 388.345x + 406.369, R^2 = 0.994; LOD = 0.78 μg/mL; LOQ = 1.97 μg/mL, peak 4^{Hs}), ferulic acid (y = 633.126x – 185.462, R^2 = 0.999, LOD = 1.85 μg/mL; LOQ = 5.61 μg/mL; peak 5^{Hs}), quercetin-3-*O*-glucoside (y = 34.843x – 160.173, R^2 = 0.991; LOD = 0.21 μg/mL; LOQ = 0.71 μg/mL; peaks 13^{Hs}, 14^{Hs}, 16^{Hs}, 19^{Hs}, 21^{Hs}, 22^{Hs}, 23^{Hs}, 25^{Hs}, 26^{Hs}, 27^{Hs}, 28^{Hs}, 29^{Hs} and 30^{Hs}) and myricetin (y = 23.287x – 581.708, R^2 = 0.999; LOD = 61.21 μg/mL; LOQ = 185.49 μg/mL; peaks 6^{Hs}, 7^{Hs}, 8^{Hs}, 9^{Hs}, 10^{Hs}, 11^{Hs}, 12^{Hs} and 24^{Hs}).

***T. mastichina* (*Tm*):** Chlorogenic acid (y = 168.823x – 161.172, R^2 = 0.999; LOD = 0.20 μg/mL; LOQ = 0.68 μg/mL, peak 1^{Tm}), caffeic acid (y = 388.345x + 406.369, R^2 = 0.994; LOD = 0.78 μg/mL; LOQ = 1.97 μg/mL, peak 2^{Tm} and 5^{Tm}), apigenin-7-*O*-glucoside (y = 10.683x – 45.794, R^2 = 0.991; LOD = 0.10 μg/mL; LOQ = 0.53 μg/mL, peak 4^{Tm}, 21^{Tm} and 32^{Tm}), taxifolin (y = 203.766x – 208.383, R^2 = 0.999; LOD = 0.67 μg/mL; LOQ = 2.02 μg/mL; peaks 6^{Tm}, 7^{Tm} and 16^{Tm}), quercetin-3-*O*-glucoside (y = 34.843x – 160.173, R^2 = 0.991; LOD = 0.21 μg/mL; LOQ = 0.71 μg/mL; peaks 8^{Tm}, 9^{Tm}, 10^{Tm}, 14^{Tm}, 15^{Tm}, 23^{Tm}, 24^{Tm}, 25^{Tm}, 26^{Tm}, 28^{Tm}, 29^{Tm} and 30^{Tm}), naringenin (y= 18.433x + 78.903, R^2 = 0.999; LOD = 0.17 μg/mL; LOQ = 0.81 μg/mL; peak 11^{Tm} and 31^{Tm}) and rosmarinic acid (y = 191.291x – 652.903, R^2 = 0.999; LOD = 0.15 μg/mL; LOQ = 0.68 μg/mL; peaks 12^{Tm}, 17^{Tm}, 18^{Tm} , 19^{Tm}, 20^{Tm} and 22^{Tm}).

***L. pedunculata* (*Lp*):** Caffeic acid (y = 388.345x + 406.369, R^2 = 0.994; LOD = 0.78 μg/mL; LOQ = 1.97 μg/mL, peak 1^{Lp}, 2^{Lp} and 6^{Lp}), *p*-coumaric acid (y = 301.950x + 6966.7, R^2 = 0.999; LOD = 0.68 μg/mL; LOQ = 1.61 μg/mL; peaks 3^{Lp}, 4^{Lp}, 5^{Lp} and 7^{Lp}), cinnamic acid ($y = 1 \times 10^6 x$ – 222.204, R^2 = 0.998; LOD = 0.33 μg/mL; LOQ = 1.19 μg/mL; peak 9^{Lp}), naringenin (y= 18.433x + 78.903, R^2 = 0.999; LOD = 0.17 μg/mL; LOQ = 0.81 μg/mL; peak 10^{Lp}), rosmarinic acid (y = 191.291x – 652.903, R^2 = 0.999; LOD = 0.15 μg/mL; LOQ = 0.68 μg/mL; peaks 11^{Lp}, 14^{Lp}, 15^{Lp} and 21^{Lp}), apigenin-7-*O*-glucoside (y = 10.683x – 45.794, R^2 = 0.991; LOD = 0.10 μg/mL; LOQ = 0.53 μg/mL, peak 16^{Lp}, 17^{Lp} and 22^{Lp}) and quercetin-3-*O*-glucoside (y = 34.843x – 160.173, R^2 = 0.991; LOD = 0.21 μg/mL; LOQ = 0.71 μg/mL; peaks 12^{Lp}, 13^{Lp}, 18^{Lp}, 19^{Lp}, 20^{Lp}, 23^{Lp} and 26^{Lp}).

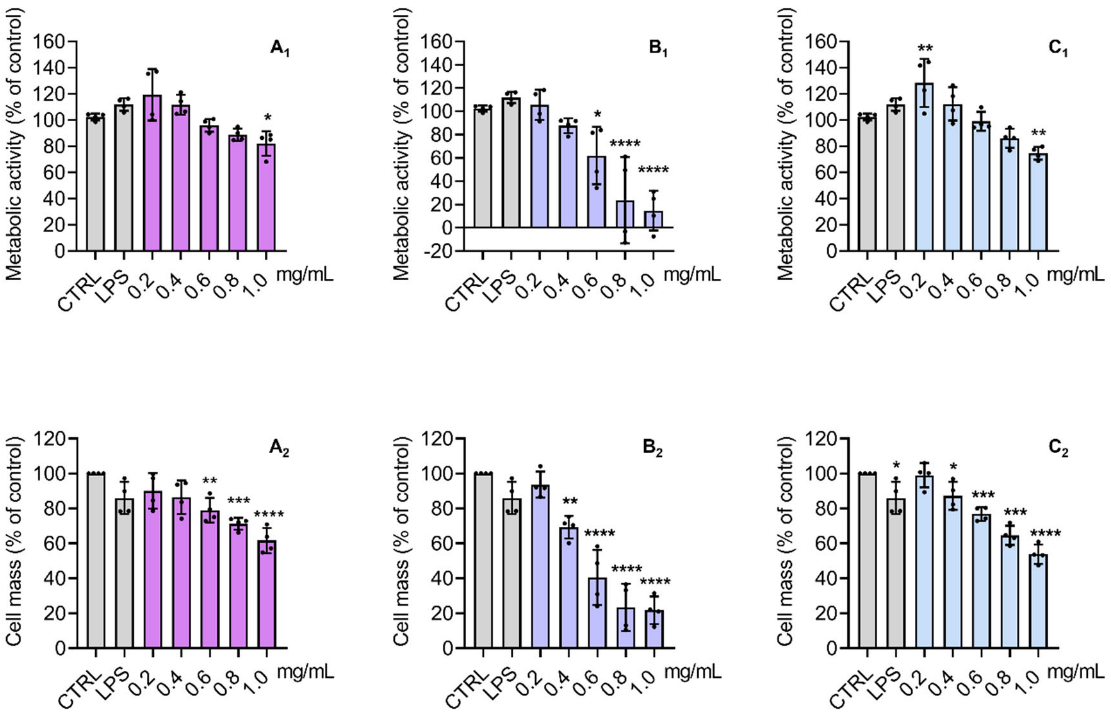

Figure S3. Effect of the HE of *H. stoechas* (A_1 and A_2), *L. pedunculata* (B_1 and B_2), *T. mastichina* (C_1 and C_2) on the metabolic activity and cell mass of Raw 264.7 macrophages, respectively. Cells were treated with HE (0.2 – 1.0 mg/mL) and LPS (0.1 µg/mL) for 24 h and metabolic activity effects were evaluated by the Alamar blue® and SRB assays. Untreated cells were used as control (CTRL). The results are expressed as percentage (%) of metabolic activity and cell mass relative to the CTRL and represent the mean ± SD of four independent experiments, each one performed in triplicates. The statistical analysis was carried out by one- ANOVA followed by Dunnett's multiple comparison test (**p*<0.05, ***p*<0.01, ****p*<0.001, and *****p*<0.0001 *versus* CTRL).
